# ACOT9, a mitochondrial metabolism-related gene, promotes ROS-associated epithelial remodeling in laryngeal squamous cell carcinoma

**DOI:** 10.1186/s12967-026-08470-x

**Published:** 2026-06-24

**Authors:** Wenwei Wang, Jian Liu, Huiqian Yang, Zheng Wang, Mengya Xie, Cailing Chen, Lingling Zhou, Xiaoming Li, Miaoqing Zhao

**Affiliations:** 1 Department of Pathology and Lab Medicine, Shandong Cancer Hospital and Institute, Shandong First Medical University and Shandong Academy of Medical Sciences, Jinan, Shandong 250117 P. R. China; 2 Department of Otolaryngology, Shandong Provincial Hospital Affiliated to Shandong First Medical University, Jinan, Shandong 250117 P. R. China; 3 Department of Otolaryngology-Head and Neck Surgery, Tongji Hospital, Tongji Medical College, Huazhong University of Science and Technology, Wuhan, Hubei P.R. China; 4 Department of Otolaryngology-Head and Neck Surgery, Huadong Hospital, Fudan University, Shanghai, China

**Keywords:** Laryngeal squamous cell carcinoma, Mitochondrial metabolism-related genes, Prognostic signature, ACOT9, Reactive oxygen species

## Abstract

**Background:**

Laryngeal squamous cell carcinoma (LSCC) is characterized by mitochondrial metabolic reprogramming, but its prognostic significance and underlying molecular mechanisms remain insufficiently understood. This study aimed to develop a prognostic mitochondrial metabolism-related genes signature (MMGS) for LSCC and to investigate the biological role of ACOT9 in tumor progression.

**Methods:**

Differentially expressed mitochondrial metabolism-related genes (MMGs) were identified from TCGA-LSCC data. A MMGS was established through machine-learning evaluation of 178 model combinations and validated in the TCGA training cohort, TCGA test cohort, and the independent GSE65858 dataset. To explore its biological relevance, immune microenvironment analysis, single-cell RNA sequencing analysis of GSE290927, scTenifoldKnk-based virtual knockout analysis, and a series of experimental assays, including immunohistochemistry, immunofluorescence, qRT-PCR, Western blotting, ROS detection, CCK-8, wound-healing, and apoptosis assays, were performed to characterize immune features, cellular heterogeneity, and the functional role of ACOT9.

**Results:**

The MMGS demonstrated excellent prognostic performance, with a C-index greater than 0.7388, and independently predicted overall survival in LSCC patients (multivariate Cox, *p* < 0.05). High-risk patients exhibited increased M0 macrophage infiltration, impaired T-cell co-stimulation, downregulated co-stimulatory molecules, and enrichment of drug metabolism-related pathways. Single-cell analysis revealed elevated MMGS scores in LSCC epithelial and myeloid cells, while ACOT9 was markedly upregulated in malignant epithelial subtypes and positively correlated with stemness (*R* = 0.18, *p* < 0.01). Virtual knockout of ACOT9 in epithelial cells identified LAMC2 and KRT17 as the two most significantly perturbed genes, and enrichment analyses indicated a downstream epithelial stress-remodeling network involving keratinization, extracellular matrix organization, inflammatory/immune signaling, and lipid inflammatory pathways. Experimental validation confirmed ACOT9 overexpression, mitochondrial colocalization with TOM20, and elevated mitochondrial ROS levels in LSCC tissues and cells. Furthermore, mitochondrial ROS scavenging or ACOT9 knockdown significantly reduced cell viability and migration while promoting apoptosis.

**Conclusions:**

MMGS is a robust and reliable prognostic tool for LSCC. ACOT9 functions as a mitochondrial-associated oncogenic factor that may promote LSCC aggressiveness through a mitochondrial ROS-dependent epithelial remodeling program, highlighting its potential as a therapeutic target for risk-stratified clinical management.

**Supplementary Information:**

The online version contains supplementary material available at 10.1186/s12967-026-08470-x.

## Introduction

Laryngeal squamous cell carcinoma (LSCC) is one of the most common malignancies of the head and neck, accounting for approximately 1–2% of all cancers worldwide [1, 2]. Despite advances in surgical techniques, radiotherapy, and chemotherapy, the 5-year overall survival rate for advanced LSCC remains unsatisfactory, ranging from 30 to 60% depending on stage at diagnosis [3, 4]. Local recurrence, lymph node metastasis, and distant spread are major contributors to poor prognosis, underscoring the urgent need for reliable prognostic biomarkers and novel therapeutic targets [5–7].

Mitochondria play a central role in cellular energy metabolism, redox homeostasis, and apoptosis regulation [8–10]. In cancer, mitochondrial metabolic reprogramming—characterized by altered oxidative phosphorylation, increased reactive oxygen species (ROS) production, and dysregulated fatty acid/amino acid metabolism—has emerged as a hallmark that supports tumor proliferation, invasion, immune evasion, and therapy resistance [11–14]. In addition to metabolic remodeling, mitochondrial perturbations may propagate through mtDNA damage and quality-control pathways, as mtDNA is particularly vulnerable to oxidative injury and cells respond through DNA repair, mitophagy, and organelle-level quality-control mechanisms [15]. Recent studies have shown that mitochondrial metabolism-related genes (MMGs) are frequently dysregulated in head and neck squamous cell carcinoma (HNSCC), including LSCC, and correlate with clinical outcomes [16, 17]. For instance, genes involved in ferroptosis suppression (FTH1) and fatty acid metabolism have been implicated in promoting tumor aggressiveness and poor survival in LSCC and HNSCC [18, 19].

Single-cell transcriptomics has further revealed cellular heterogeneity in LSCC, highlighting mitochondrial reprogramming in malignant epithelial cells and immune cell subsets within the tumor microenvironment (TIME). Single-cell RNA sequencing provides an opportunity to dissect cell-type-specific regulatory relationships that may be obscured in bulk transcriptomic data, thereby supporting its application for inferring epithelial-state-specific downstream networks in LSCC [20]. However, systematic integration of bulk RNA-seq, single-cell analysis, machine learning-based prognostic modeling, immune landscape characterization, and functional validation of key mitochondrial metabolism-related genes (MMGs) remains limited in LSCC.

In this study, we aimed to: (1) identify differentially expressed MMGs and construct a robust prognostic mitochondrial metabolism-related genes signature (MMGS) using multi-algorithm machine learning; (2) evaluate its association with immune infiltration, pathway enrichment, and drug sensitivity; (3) explore cellular heterogeneity via single-cell RNA-seq; and (4) experimentally validate the functional role of a prominent hub gene, ACOT9, in LSCC progression. Our findings provide new insights into mitochondrial-driven mechanisms in LSCC and offer a potential tool for risk stratification and targeted therapy.

## Methods

### Data collection and preprocessing

RNA sequencing (RNA-seq) data and corresponding clinical annotations for patients with LSCC were downloaded from The Cancer Genome Atlas (TCGA) (https://portal.gdc.cancer.gov/) database on August 08, 2025. Cases lacking survival information were excluded prior to analysis. Ultimately, transcriptomic profiles from 111 LSCC tumor tissues and 12 matched adjacent non-tumorous tissues were included, along with the clinical data of the 111 eligible patients. In addition, the GSE65858 dataset was retrieved from the Gene Expression Omnibus (GEO) database as an external validation cohort. Because GSE65858 comprises 270 patients with head and neck squamous cell carcinoma (HNSCC) rather than a dedicated LSCC cohort, LSCC cases were extracted according to the annotated primary tumor site. Specifically, only 48 patients with LSCC were identified in this dataset, and all of them had complete overall survival (OS) information as well as available transcriptomic and clinical data; therefore, these 48 cases were included as the external validation cohort. The GSE130605 dataset was additionally retrieved from the GEO database and used as an independent expression validation dataset. GSE130605 contains LSCC tumor tissues and corresponding control tissues, and was used to validate the expression patterns of the model genes between LSCC and control samples. This dataset was used only for expression validation rather than survival-based prognostic validation.

### Identification of differentially expressed genes (DEGs) by differential expression analysis and key co-expression modules by weighted gene co-expression network analysis (WGCNA)

Differential expression analysis between adjacent non-tumorous tissues and LSCC tumor tissues was performed using the limma R package. Genes meeting the thresholds of |log2 fold change (FC)| ≥0.585 and adjusted *p* < 0.05 were defined as DEGs. WGCNA was subsequently conducted using the WGCNA R package to identify disease-associated gene modules and hub genes [21]. After preprocessing and filtering of the RNA-seq data, a gene co-expression similarity matrix was generated, and a scale-free network was constructed using a soft-thresholding power of β = 16, which achieved a scale-free topology fit index of R^2^ > 0.90. Modules showing the strongest correlations with LSCC were then selected for downstream identification of disease-relevant genes.

### Acquisition of MMGs and identification of candidate hub genes

MMGs were obtained from the MitoCarta 3.0 database (https://www.broadinstitute.org/mitocarta/mitocarta30-inventory-mammalian-mitochondrial-proteins-and-pathways), the GSEA resource (http://www.gsea-msigdb.org/gsea/index.jsp), and relevant published literature [22, 23]. Candidate mitochondrial metabolism-related hub genes were identified by intersecting DEGs, genes derived from LSCC-associated key modules identified by WGCNA, and MMGs. The shared genes were identified using the VennDiagram R package, followed by univariate prognostic analysis in the TCGA cohort. Genes significantly associated with overall survival were retained for downstream analyses.

### Machine learning–based construction of the prognostic model

A total of 111 patients with LSCC were randomly divided into a training cohort and a testing cohort at a ratio of 6:4. To develop a prognostic model with optimal robustness, discrimination, and stability, 10 machine learning algorithms were integrated to generate 178 algorithmic combinations. These algorithms included Lasso, Elastic Net, Ridge, stepwise Cox regression, CoxBoost, partial least squares regression for Cox models (plsRcox), random survival forest (RSF), SuperPC, generalized boosted regression modeling (GBM), and survival support vector machine (survival-SVM). Model performance was evaluated using the concordance index (C-index), and the resulting models were ranked accordingly. All 178 combinations were first trained in the training cohort and then validated in the testing cohort and 48 LSCC cases in GSE65858 to identify the model with the strongest prognostic value for overall survival (OS).

### Validation of the prognostic signature

To assess the prognostic significance of the signature, a risk score was calculated for each patient in the TCGA cohort using the formula: risk score = $\mathop \sum\nolimits_{i = 1}^n \left( {{\rm{geneexpression*regressioncoefficient}}} \right)$. Patients were classified into high- and low-risk groups according to the median risk score of the training cohort. Kaplan–Meier survival analysis was performed using the survival and survminer R packages, with differences in OS evaluated by the log-rank test. The predictive performance of the signature was further assessed by time-dependent ROC analysis using the timeROC R package. The model was validated in the training, testing, and entire TCGA cohorts, as well as in the external 48 LSCC cases in GSE65858 cohort. Correlation analysis was subsequently conducted between the risk score and target gene expression. Moreover, univariate and multivariable Cox regression analyses were performed in the training, testing, and entire TCGA cohorts to determine whether the prognostic signature served as an independent predictor of survival. A nomogram integrating prognostic variables was constructed to estimate survival probabilities, and calibration curves were used to evaluate its predictive agreement.

### Gene set enrichment analysis (GSEA)

To characterize the signaling pathways distinguishing the high- and low-risk groups, GSEA was performed using the clusterProfiler R package with the “c2.cp.kegg_legacy.v2024.1.Hs.symbols” gene set obtained from the MSigDB database. Tumor-associated pathways showing significant enrichment in either group were identified and displayed, using *p* < 0.05 as the significance cutoff.

### Assessment of immune infiltration and the tumor microenvironment

To investigate the immune landscape of LSCC, immune cell infiltration patterns between the high- and low-risk groups were analyzed using the CIBERSORT algorithm [24, 25], a deconvolution-based approach for estimating the relative fractions of immune cell populations from bulk transcriptomic data. The proportions of 22 immune cell types (including seven T cell types, naive and memory B cells, plasma cells, and NK cells) were calculated for each sample. Meanwhile, ssGSEA was conducted using the GSVA R package to quantify the enrichment scores of immune cell populations and immune-related pathways in both groups [26, 27]. Furthermore, the TIDE algorithm was employed to predict differences in potential response to immune checkpoint inhibition between the two subgroups [28].

### Prediction of therapeutic response to targeted agents and chemotherapeutic drugs

To explore potential individualized treatment strategies for patients with LSCC, drug sensitivity was evaluated based on the risk score. The half-maximal inhibitory concentration (IC50) of candidate targeted agents and chemotherapeutic drugs was estimated using the oncoPredict R package in combination with genomic drug response data obtained from the Genomics of Drug Sensitivity in Cancer (GDSC) database (https://www.cancerrxgene.org/) [27].

### Processing and annotation of single-cell RNA-seq data

The scRNA-seq dataset GSE290927 for LSCC was obtained from the GEO database. Cells passing quality control were retained for downstream analysis, including those with more than 200 detected genes and genes expressed in at least three cells. Further filtering was performed using the following thresholds: nFeature_RNA of 500–7,000, nCount_RNA of 500– 60,000, percent.mt < 20%, and percent.rb < 40%. The filtered cells were then normalized using the LogNormalize method and scaled by linear regression. The top 2,000 highly variable genes were identified using the FindVariableFeatures function. To remove batch effects, Harmony integration was applied prior to dimensionality reduction. PCA was subsequently performed, followed by UMAP for low-dimensional visualization. Cell clusters were identified using the FindNeighbors and FindClusters functions at a resolution of 1.2. Cell identities were assigned based on SingleR annotation together with canonical marker genes, cluster-enriched genes, and established cell-type-specific expression profiles. The activity of hub genes across distinct cell types was subsequently evaluated using AUCell, AddModuleScore, and UCell scoring methods.

### Shapley additive explanations (SHAP)-based prioritization of weighted hub genes

To further prioritize hub genes with greater predictive importance, SHAP analysis was performed based on the optimal machine-learning model. SHAP values were calculated for each hub gene across all samples to quantify its contribution to the model output. Feature importance was then ranked according to the mean absolute SHAP value. Hub genes with higher SHAP values were considered to have greater influence on model prediction and were therefore defined as weighted hub genes for subsequent analyses. In addition, SHAP summary and dependence analyses were used to further characterize the overall impact and directionality of hub gene expression on the predictive model.

### Virtual knockout analysis using scTenifoldknk

To explore the downstream regulatory perturbations associated with ACOT9 loss at the single-cell level, virtual knockout analysis was performed using the scTenifoldKnk R package in epithelial cells derived from the LSCC single-cell RNA-seq dataset [29]. The raw count matrix was extracted from the Seurat object, and the top variable genes were selected for network construction. ACOT9 was specified as the knockout gene, and the scTenifoldKnk framework was applied according to the recommended workflow to infer differential regulatory changes after in silico gene deletion. The resulting diffRegulation matrix was used to calculate fold change (FC), Z score, and adjusted q value for each gene. Genes with |Z| ≥2 and q < 0.01 were considered significantly perturbed genes. Differentially perturbed genes were visualized using FC-ranked bar plots and Z score versus −log10(q value) scatter plots.

### Gene ontology (GO) and Kyoto encyclopedia of genes and genomes (KEGG) enrichment analyses of perturbed genes

To characterize the biological functions and pathways associated with ACOT9 perturbation, functional enrichment analyses were performed on the top 100 perturbed genes ranked by Z score derived from the scTenifoldKnk results. GO enrichment analysis, including biological process (BP), cellular component (CC), and molecular function (MF), as well as KEGG pathway enrichment analysis, were conducted using the clusterProfiler R package with org.Hs.eg.db as the annotation reference. Terms or pathways with adjusted *p* < 0.05 were considered significantly enriched [30].

### Clinical tissue collection and histological validation

Paired tumor tissues and adjacent non-tumorous tissues were collected from six patients with LSCC who underwent surgical resection. None of the patients had received preoperative radiotherapy, chemotherapy, or other antitumor treatment before surgery. All tissue specimens were confirmed by postoperative pathological examination. Tissue samples were fixed in 4% paraformaldehyde, embedded in paraffin, and sectioned at 4-μm thickness for subsequent immunohistochemistry (IHC) and immunofluorescence (IF) analyses.

### Immunohistochemistry

Paraffin-embedded tissue sections were deparaffinized in xylene and rehydrated through a graded ethanol series. Antigen retrieval was performed in citrate buffer (pH 6.0) using heat-induced epitope retrieval. Endogenous peroxidase activity was blocked with 3% hydrogen peroxide, followed by blocking with 5% goat serum for 1 h at room temperature to reduce nonspecific binding. The sections were then incubated overnight at 4 °C with an anti-ACOT9 primary antibody (Proteintech, China; Cat No. 15,901–1-AP; rabbit polyclonal IgG; dilution: 1:500). After washing, the sections were incubated with an HRP-conjugated Goat Anti-Rabbit IgG (H+L) secondary antibody (ABclonal, Cat. No.AS014; dilution: 1:200) for 30 min at room temperature. Subsequently, the sections were stained using a Diaminobenzidine kit (ZSGB-BIO, China). All images were observed and acquired with an optical microscope (EVOS M7000).

### Immunofluorescence staining

For IF staining, paraffin sections were deparaffinized, rehydrated, and subjected to antigen retrieval using citrate buffer (pH 6.0). After blocking with 5% bovine serum albumin for 1 h at room temperature, the sections were incubated overnight at 4 °C with primary antibodies against ACOT9 (ABclonal, China, Cat. No.A15416; rabbit polyclonal antibody; dilution: 1:200) and TOM20 (Proteintech, China, Cat No. 66,777–1-Ig; mouse monoclonal antibody; dilution: 1:400). After washing with PBS, the sections were incubated with the appropriate Alexa Fluor 488- or Alexa Fluor 647-conjugated secondary antibodies (ABclonal; China, dilution: 1:200) for 1 h at room temperature in the dark. Nuclei were counterstained with DAPI for 10 min. Fluorescence images were captured using a Zeiss LSM900 confocal microscope to evaluate the expression patterns and distribution of ACOT9 and TOM20 in LSCC and adjacent non-tumorous tissues.

### Cell culture, MitoQ treatment, and siRNA transfection

The LSCC cell line LCC and the human immortalized keratinocyte cell line HaCaT were obtained from Wuhan Pricella Biotechnology Co., Ltd. (Wuhan, Hubei, China). HaCaT cells were maintained in DMEM, whereas LCC cells were cultured in RPMI-1640 medium (Gibco, Grand Island, NY, USA), both supplemented with 10% heat-inactivated fetal calf serum (FCS; Gibco), 10 IU/mL penicillin, and 10 μg/mL streptomycin (Servicebio Technology, Wuhan, China). Cells were maintained at 37 °C in a humidified atmosphere containing 5% CO₂. For siRNA-mediated knockdown, cells were transfected with the indicated siRNAs for 6 h. The transfection medium was then removed, cells were washed with PBS, and complete medium was added. After an additional 24, 48, or 72 h of culture, cell viability, migration, and apoptosis were assessed, while gene expression levels were determined at the 24-h time point. The siRNA sequences were as follows: siACOT9 antisense, 5′-ATGGAAGAAAGGAAATTACTTC-3′.

### Wound-healing assay

Cell migratory capacity was assessed using a wound-healing assay. Briefly, cells were seeded into culture plates and allowed to reach an appropriate confluence. A scratch was then created across the cell monolayer using a 10-μL pipette tip. After 48 h of incubation, detached cells were gently removed by washing with PBS, and images were captured under a microscope at × 4 magnification. The wound area was quantified using image analysis software, and the extent of cell migration was evaluated based on the change in wound area.

### Quantitative real-time PCR

Gene expression was quantified by quantitative real-time PCR (qRT-PCR). Total RNA was extracted from cells and reverse-transcribed into complementary DNA (cDNA). qRT-PCR was then performed using gene-specific primers and the SYBR Premix Ex Taq kit (TaKaRa Biotechnology, USA) according to the manufacturer’s instructions. The primer sequences were as follows: “ACOT9 forward, 5′-ATCCACTCCGCCAAGATGTC-3′, and reverse, 5′-GTCTTCCCGACCCAGCTAAC-3′; β-actin forward, 5′-CATGTACGTTGCTATCCAGGC-3′, and reverse, 5′-CTCCTTAATGTCACGCACGAT-3′”. Amplification specificity was verified by melting curve analysis. Relative gene expression levels were normalized to β-actin and calculated using the 2^^−ΔΔCt^ method.

### Western blot analysis

LCC and HaCaT cells were lysed in RIPA buffer containing protease inhibitors to extract total protein. Protein concentrations were measured using a BCA protein quantification kit. Equal amounts of protein from each sample were separated by SDS-PAGE and subsequently transferred onto PVDF membranes. The membranes were blocked with 5% non-fat milk in TBST for 1 h at room temperature and then incubated overnight at 4 °C with a primary antibody against ACOT9 (Proteintech, China; Cat No. 15,901–1-AP; rabbit polyclonal IgG; dilution: 1:500) and β-actin. After washing with TBST, the membranes were incubated with the appropriate HRP-conjugated secondary antibodies for 1 h at room temperature. Immunoreactive bands were detected using an ECL reagent, and images were acquired using a gel imaging system. β-actin served as the loading control.

### Cell viability assay

Cell viability was determined using the CCK-8 assay (MedChemExpress, New Jersey, USA). LCC cells were seeded in 96-well plates and cultured for 12 h. The cells were then treated with MitoQ (200 nM; Shanghai Yuanye Bio-Technology Co., Ltd., China) or subjected to siACOT9 transfection. Cell viability was evaluated at 24, 48, and 72 h after treatment. Subsequently, 10 μL of CCK-8 reagent was added to each well, and the cells were incubated for an additional 2 h at 37 °C in a humidified atmosphere containing 5% CO₂. The optical density was measured at 450 nm.

### Flow cytometry

Cell apoptosis was assessed by flow cytometry using an Annexin V-FITC/PI Apoptosis Kit (Elabscience Biotechnology Co., Ltd., China; Cat No. E-CK-A211) according to the manufacturer’s instructions. Briefly, treated cells were collected, washed, and stained with Annexin V-ABflo® 488 and PI. The stained cells were subsequently analyzed using a BD flow cytometer (BD Biosciences, San Jose, CA, USA). During acquisition, debris, dead cells, and cell aggregates were excluded by forward scatter and side scatter gating. Data were analyzed using FlowJo software (Tree Star, Ashland, OR, USA).

### MitoSOX red staining and analysis

Mitochondrial superoxide levels were assessed using the MitoSOX Red fluorescent probe (MCE, China). LCC and HaCaT cells were seeded in six-well plates for flow cytometric analysis or on confocal dishes for fluorescence imaging at an appropriate density and cultured under standard conditions until reaching 80–90% confluence. The culture medium was then removed, and the cells were washed three times with PBS. Subsequently, cells were incubated with 5 μM MitoSOX Red at 37 °C for 10 min in the dark. For fluorescence imaging, nuclei were counterstained with Hoechst and images were captured using a confocal microscope. For flow cytometric analysis, stained cells were collected and analyzed using a flow cytometer (Thermo Fisher, USA), and the data were processed using FlowJo software. Mitochondrial ROS levels were evaluated based on the fluorescence intensity of MitoSOX Red.

### Statistical analysis

Statistical analyses were performed using R software (version 4.4.0) and GraphPad Prism 8.0 (GraphPad Software, La Jolla, CA, USA). Comparisons between two paired groups or ordinal variables were conducted using the Wilcoxon test. Spearman correlation analysis was used to assess the strength and direction of linear associations between continuous variables. Survival analysis was performed using the Kaplan–Meier method, and differences between groups were assessed by the log-rank test. Univariate and multivariable Cox regression analyses were conducted to identify prognostic factors and evaluate their independent prognostic significance. Receiver operating characteristic (ROC) curve analysis was used to assess the predictive performance of the risk score. Data normality was examined using the Kolmogorov–Smirnov test. A two-sided *p* < 0.05 was considered statistically significant.

## Results

### Identification of DEGs, WGCNA module–trait associations in LSCC, and prognostic MMGs

**Fig. 1:**
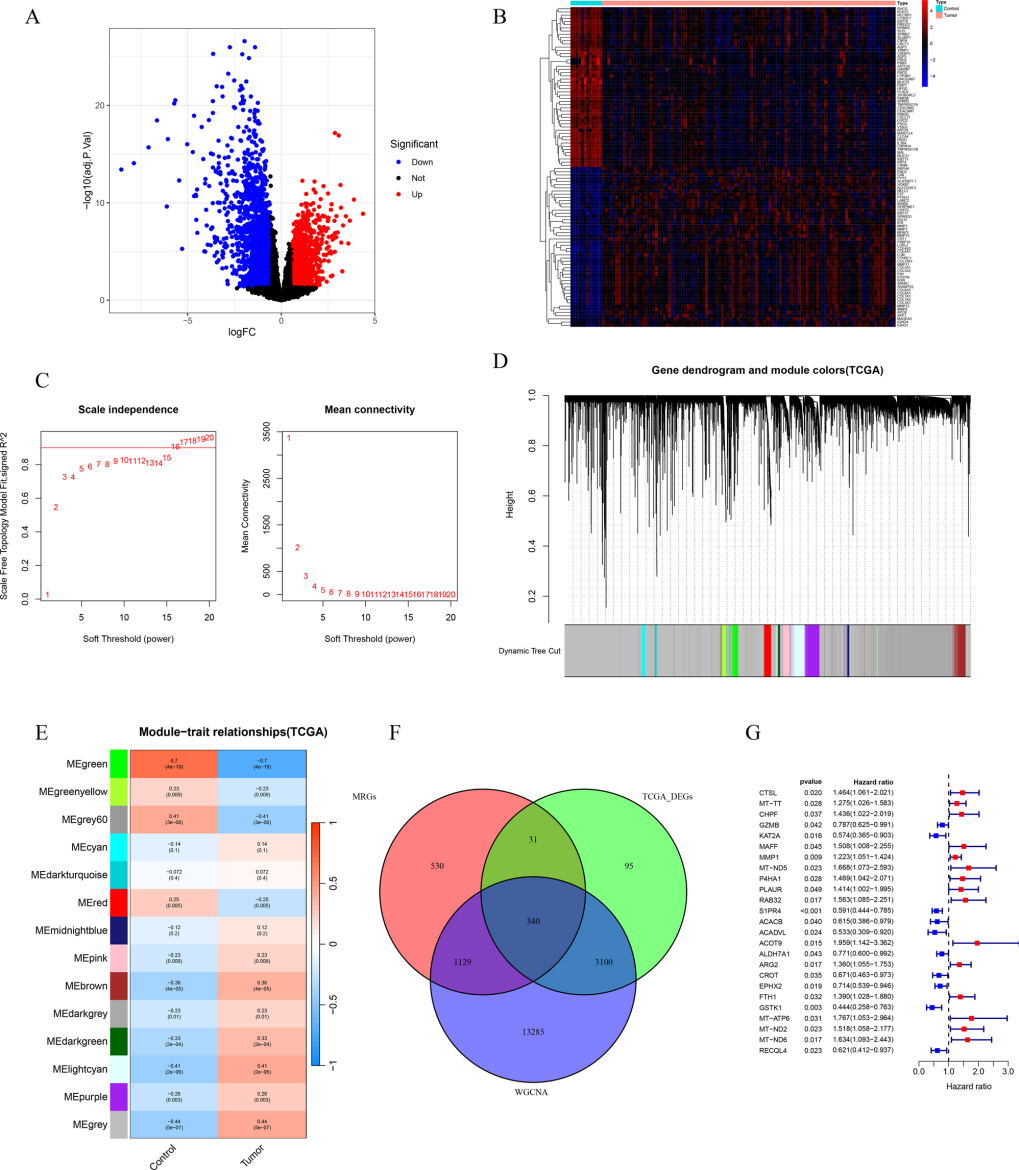
Identification of differentially expressed MMGs and WGCNA module–trait associations in LSCC. (**A**) The volcano plot shows the distribution of DEGs between LSCC and adjacent normal tissues. (**B**) Heatmap illustrating the expression patterns of significantly dysregulated genes in LSCC compared with control tissues. (**C**) Determination of the soft-thresholding power (β) in the WGCNA. Analysis of the scale-free topology fit index (R^2^) for different soft-thresholding powers. A power of β = 16 (red point) was selected where R^2^ approaches 0.9, indicating the network approximates a scale-free topology, and mean connectivity under various soft-thresholding powers. The selected β = 16 corresponds to a point where mean connectivity stabilizes. (**D**) Gene clustering dendrogram based on topological overlap matrix dissimilarity. Dynamic tree cutting identified distinct co-expression modules, each represented by a unique color. (**E**) Module–trait relationships. Heatmap displaying correlations between module eigengenes (MEs) and clinical traits (LSCC vs. control). Positive correlations are depicted in red and negative correlations in blue, with color intensity indicating correlation strength and *p*-values shown in parentheses. (**F**) Venn diagram of overlapping genes among WGCNA module genes, MMGs, and DEGs. (**G**) Univariate Cox regression analysis of mitochondrial differentially expressed genes associated with LSCC prognosis. The forest plot displays hazard ratios (HRs) with 95% confidence intervals and *p*-values for each mitochondrial gene, highlighting genes with significant prognostic value. (*p < 0.05, **p < 0.01, ***p < 0.001). Abbreviations: MMGs: mitochondrial metabolism-related genes; WGCNA: weighted gene co-expression network analysis: LSCC; laryngeal squamous cell carcinoma; DEGs: differentially expressed genes

We first identified 3,566 DEGs in LSCC by comparing tumor tissues with adjacent normal tissues. The volcano plot revealed a distinct distribution of DEGs, with significantly upregulated and downregulated genes (Fig. 1A). A heatmap further confirmed the expression profiles of these significantly dysregulated genes between LSCC and control tissues (Fig. 1B). Subsequently, WGCNA was performed to construct co-expression networks. To achieve scale-free topology, a soft-thresholding power (β) of 16 was selected, where the scale-free fit index (R^2^) approached 0.9 and mean connectivity stabilized (Fig. 1C and D). Dynamic tree cutting of the gene clustering dendrogram based on topological overlap matrix (TOM) dissimilarity identified multiple distinct co-expression modules, each assigned a unique color (Fig. 1E). Module–trait correlation analysis revealed that multiple co-expression modules were significantly associated with LSCC (*p* < 0.05), including MEgreen, MEgreenyellow, MEgrey60, MEcyan, MEred, MEpink, MEbrown, MEdarkgrey, MEdarkgreen, MElightcyan, MEpurple, and MEgrey. Among these, the MEgreen module eigengene showed a strong negative correlation with LSCC (*R* = −0.70, *p* = 4 × 10^−19) (Fig. 1E), whereas the MEgrey module eigengene exhibited a significant positive correlation (*R* = 0.44, *p* = 5 × 10^−7). Based on the significantly LSCC-associated modules, a total of 17,854 genes were extracted as WGCNA module genes. These genes were subsequently intersected with 2,030 MMGs and 3,566 DEGs, yielding 340 overlapping genes. Finally, univariate Cox regression analysis was performed in the TCGA LSCC cohort to evaluate the prognostic significance of these 340 candidate genes (Fig. 1F). The results revealed 10 low-risk genes (protective, HR < 1) and 15 high-risk genes (hazardous, HR > 1), as illustrated in the forest plot with hazard ratios, 95% confidence intervals, and *p*-values (Fig. 1G).

### Construction and evaluation of machine-learning–based prognostic risk models for LSCC

**Fig. 2:**
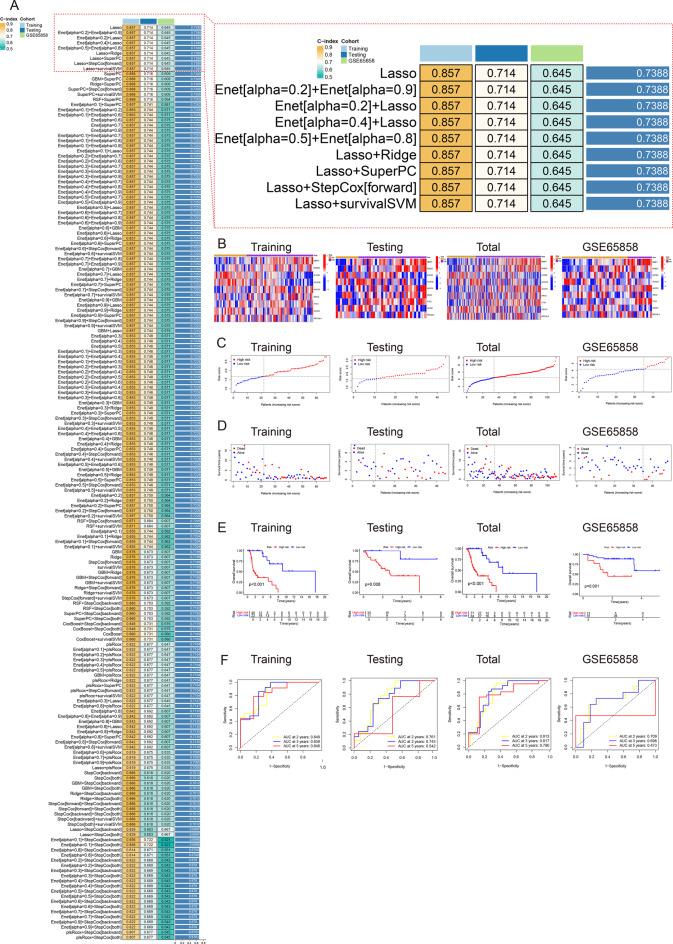
Construction and evaluation of machine-learning–based prognostic MMGS for LSCC. (**A**) A total of 178 combinations of 10 machine learning algorithms were applied to construct prognostic risk models for LSCC patients. Patients were randomly divided into training and test cohorts at a 6:4 ratio. For each model, the concordance index (C-index) was calculated across the TCGA training set, TCGA test set, and external validation dataset (GSE65858). The top-performing models consistently exhibited C-index values above 0.7388. (**B**) Heatmap showing the expression patterns of 9 hub genes across high-risk and low-risk subgroups in the training, testing, full TCGA cohorts, and GSE65858 validation cohort. Red and blue represent higher and lower expression levels, respectively. (**C**) Correlation analysis between the risk score and the prognostic risk of LSCC patients, demonstrating that higher risk scores are associated with increased mortality probability across all datasets. (**D**) Distribution of risk scores and corresponding survival status. Patients sorted by increasing risk score reveal a pattern in which high-risk patients (red) exhibit higher mortality rates compared with low-risk patients (blue). (**E**) Kaplan–Meier survival curves comparing the overall survival between high-risk and low-risk groups in the TCGA training group, test group, total cohort, and GSE65858 dataset. High-risk groups consistently showed significantly reduced survival (*p* < 0.05). (**F**) Time-dependent ROC curves evaluating the predictive accuracy of the risk model for 2-, 3-, and 5-year survival in each dataset. (*p < 0.05, **p < 0.01, ***p < 0.001). Abbreviations: MMGS: mitochondrial metabolism-related genes signature; LSCC; laryngeal squamous cell carcinoma

To develop a robust prognostic risk model for LSCC, we systematically evaluated 178 combinations of 10 machine learning algorithms using the prognostic MMGs identified previously. Patients from the TCGA-LSCC cohort were randomly divided into training and test sets at a 6:4 ratio, with 48 LSCC cases in GSE65858 dataset serving as an independent external validation cohort. For each constructed model, the concordance index (C-index) was computed in the TCGA training set, TCGA test set, and 48 LSCC cases in GSE65858 validation set. Several models demonstrated superior performance, consistently achieving C-index values exceeding 0.7388 across all datasets, indicating excellent discriminatory ability for survival prediction (Fig. 2A). To further evaluate the robustness of the optimal machine learning model in prognostic prediction for LSCC, patients were classified into high-risk and low-risk groups according to the median risk score derived from the training set. The risk score was calculated as follows: Risk score = (−0.43955 * ACACB) + (0.562686 * ACOT9) + (0.205275 * ARG2) + (0.684833 * FTH1) + (−0.78463 * GSTK1) + (0.410209 * MMP1) + (0.252956 * RAB32) + (−0.22743 * RECQL4) + (−0.26943 * S1PR4). A heatmap illustrated the expression patterns of these 9 hub genes, revealing clear separation between high-risk and low-risk subgroups in the TCGA training, test, entire TCGA cohorts, and 48 LSCC cases in GSE65858 validation cohort, with red denoting higher expression and blue lower expression (Fig. 2B). Risk scores were calculated for each patient using the coefficients derived from the optimal machine learning model. Correlation analysis showed a strong positive association between the risk score and prognostic risk. The results indicated that the risk score was significantly increased in the high-risk group (Fig. 2C). When patients were ordered by ascending risk score, the distribution of survival status demonstrated that patients at high risk had shorter survival, confirming the model’s ability to stratify patients effectively (Fig. 2D). Kaplan–Meier survival analysis further validated the prognostic power of the risk model. In the TCGA training group, test group, entire cohort, patients in the high-risk group exhibited significantly poorer overall survival than those in the low-risk group (all log-rank *p* < 0.05), highlighting consistent separation between risk strata (Fig. 2E). Time-dependent ROC curves were generated to assess the model’s predictive accuracy for 2-, 3-, and 5-year overall survival. The results showed that the area under the curve (AUC) was highest for the 5-year overall survival curve (Fig. 2F).

### Evaluation for predicting the prognosis of LSCC

**Fig. 3:**
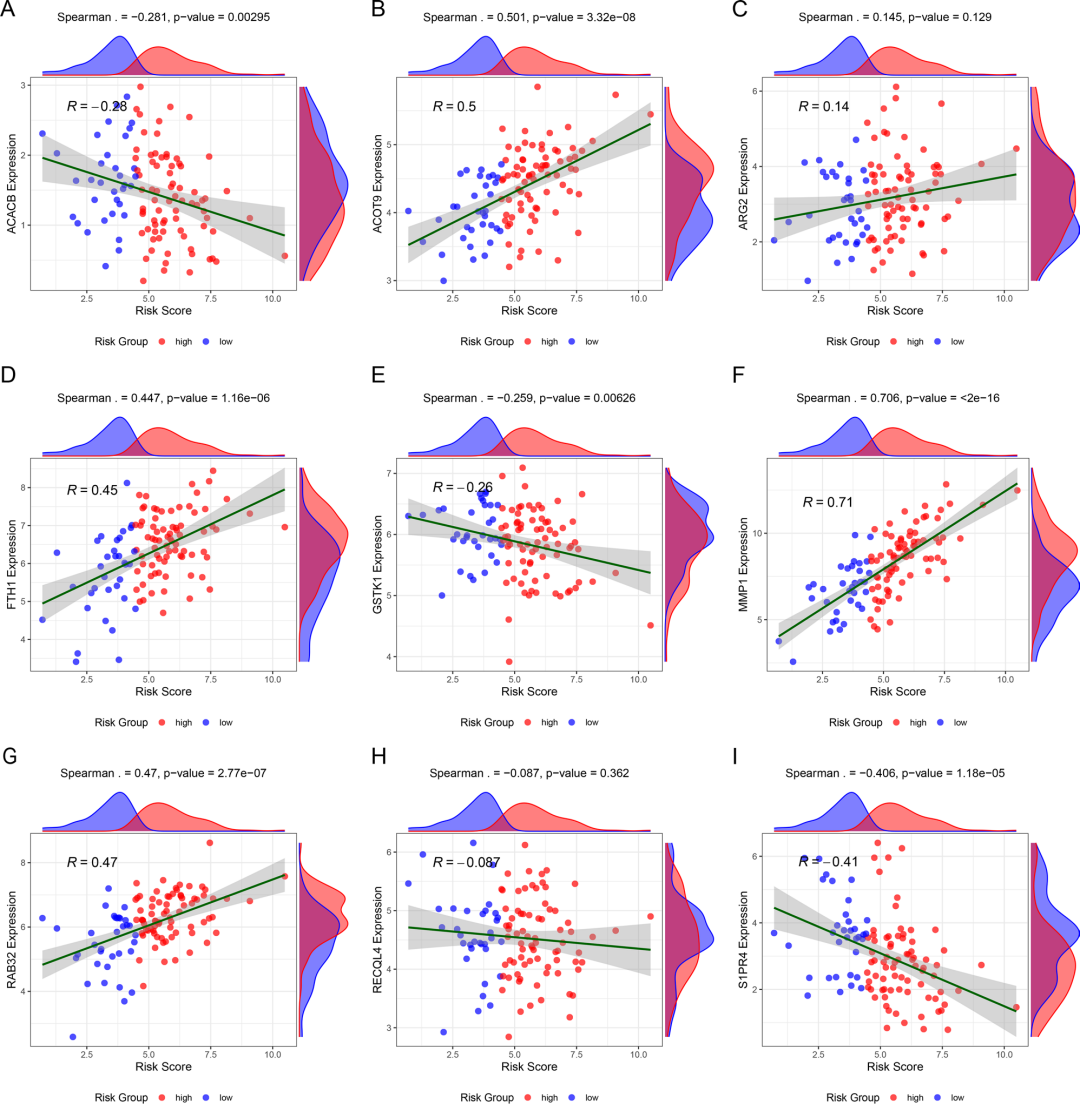
Correlations of nine MMGs with the MMGS. Scatter plots (**A-I**) showing the correlations between the MMGS risk score and the expression levels of the nine model genes in LSCC patients. Patients were stratified into high-risk and low-risk groups. Each panel corresponds to one gene (ACACB, ACOT9, ARG2, FTH1, GSTK1, MMP1, RAB32, RECQL4, and S1PR4) and displays the Spearman correlation coefficient and the associated *p*-value. Abbreviations: MMGs: mitochondrial metabolism-related genes; MMGS: mitochondrial metabolism-related genes signature

**Fig. 4:**
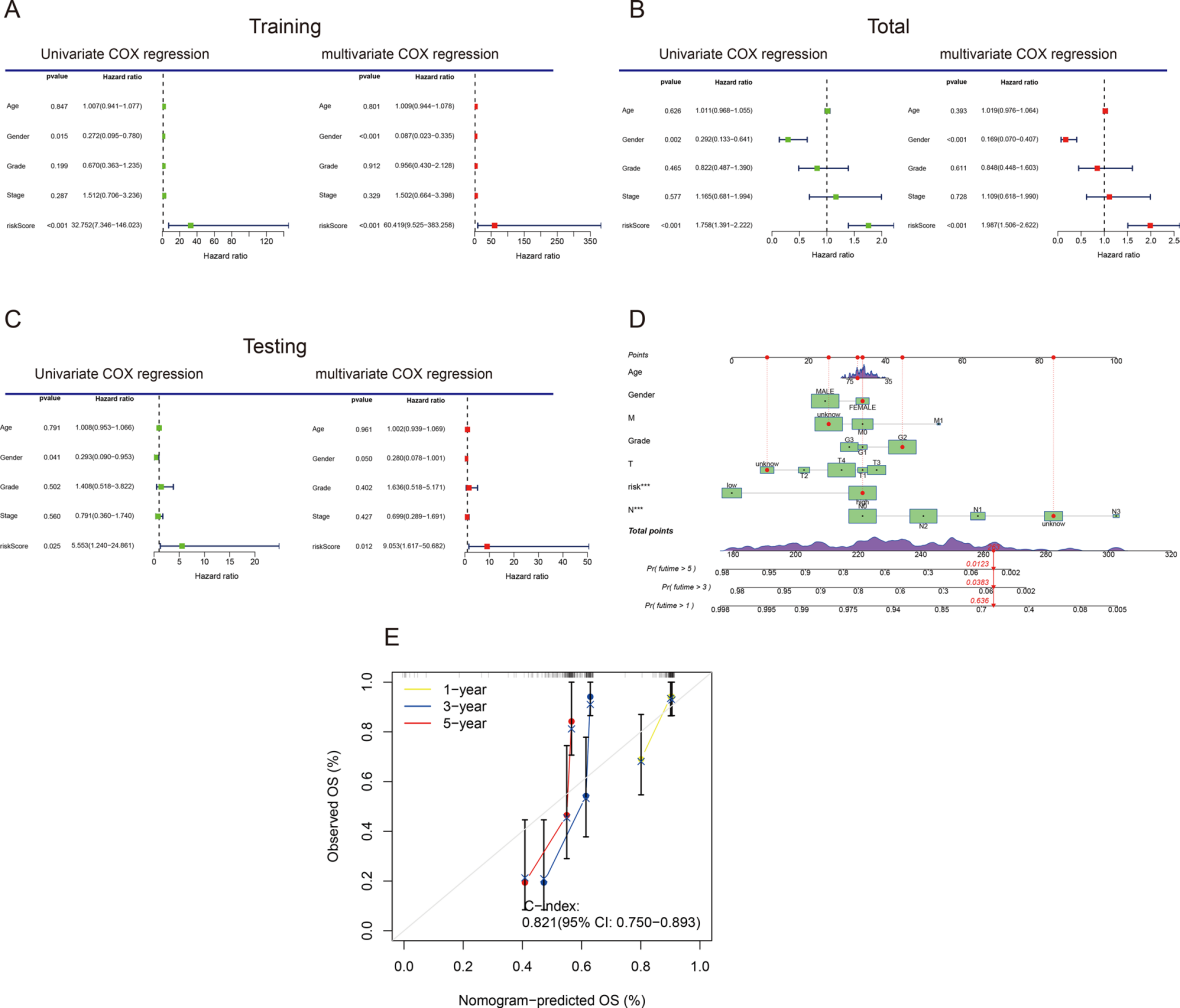
Univariate and multivariate Cox regression analyses and nomogram evaluation for predicting the prognosis of LSCC. (**A–C**) Univariate and multivariate Cox regression analyses evaluating the prognostic value of clinical features and the MMGS risk score in the training cohort (**A**), total cohort (**B**), and testing cohort (**C**). (**D**) A nomogram integrating the MMGS risk score with key clinical factors (including age, gender, grade, T stage, and N stage) to predict the 1-, 3-, and 5-year overall survival probabilities of LSCC patients. (**E**) Calibration curves assessing the predictive performance of the nomogram for 1-, 3-, and 5-year overall survival. Abbreviations: LSCC; laryngeal squamous cell carcinoma: MMGS: mitochondrial metabolism-related genes signature

To investigate the relationship between the individual components of the MMGs and the calculated risk scores, we performed Spearman correlation analyses for each of the 9 hub MMGs (ACACB, ACOT9, ARG2, FTH1, GSTK1, MMP1, RAB32, RECQL4, and S1PR4) in LSCC patients stratified by high-risk and low-risk groups. Scatter plots demonstrated the correlations between the MMGS and the expression levels of each gene (Fig. 3A–I). Among these 9 genes, ACOT9, ARG2, FTH1, RAB32, and MMP1 are positively correlated with the the MMGS, while ACACB, GSTK1, RECQL4, and S1PR4 are negatively correlated with the MMGS. Next, we conducted univariate and multivariate Cox regression analyses to assess whether the MMGS served as an independent prognostic factor, beyond conventional clinical parameters. In the training cohort, univariate Cox regression identified the MMGS along with several clinical features (including age, grade, T stage, and N stage) as significantly associated with overall survival; multivariate analysis further confirmed that the MMGS remained an independent predictor of prognosis (hazard ratio with 95% confidence interval and *p*-value; Fig. 4A). Similar results were observed in the total TCGA cohort (Fig. 4B) and the testing cohort (Fig. 4C), where the MMGS consistently demonstrated independent prognostic value (all *p* < 0.05 in multivariate models), highlighting its robustness across different patient subsets. To facilitate clinical application, we constructed a nomogram that integrated the MMGS risk score with key clinical factors (age, gender, grade, T stage, and N stage) to predict 1-, 3-, and 5-year overall survival probabilities for LSCC patients (Fig. 4D). Each variable was assigned points based on its regression coefficient, allowing individualized risk assessment by summing the points and mapping to the survival probability scales. Calibration curves for the nomogram showed excellent agreement between the predicted and actual 1-, 3-, and 5-year overall survival rates across the cohorts, with the calibration lines closely approximating the ideal 45-degree reference line, indicating high predictive accuracy and reliability of the combined model (Fig. 4E).

### Therapeutic implications associated with the MMGS and immune microenvironment landscape

**Fig. 5:**
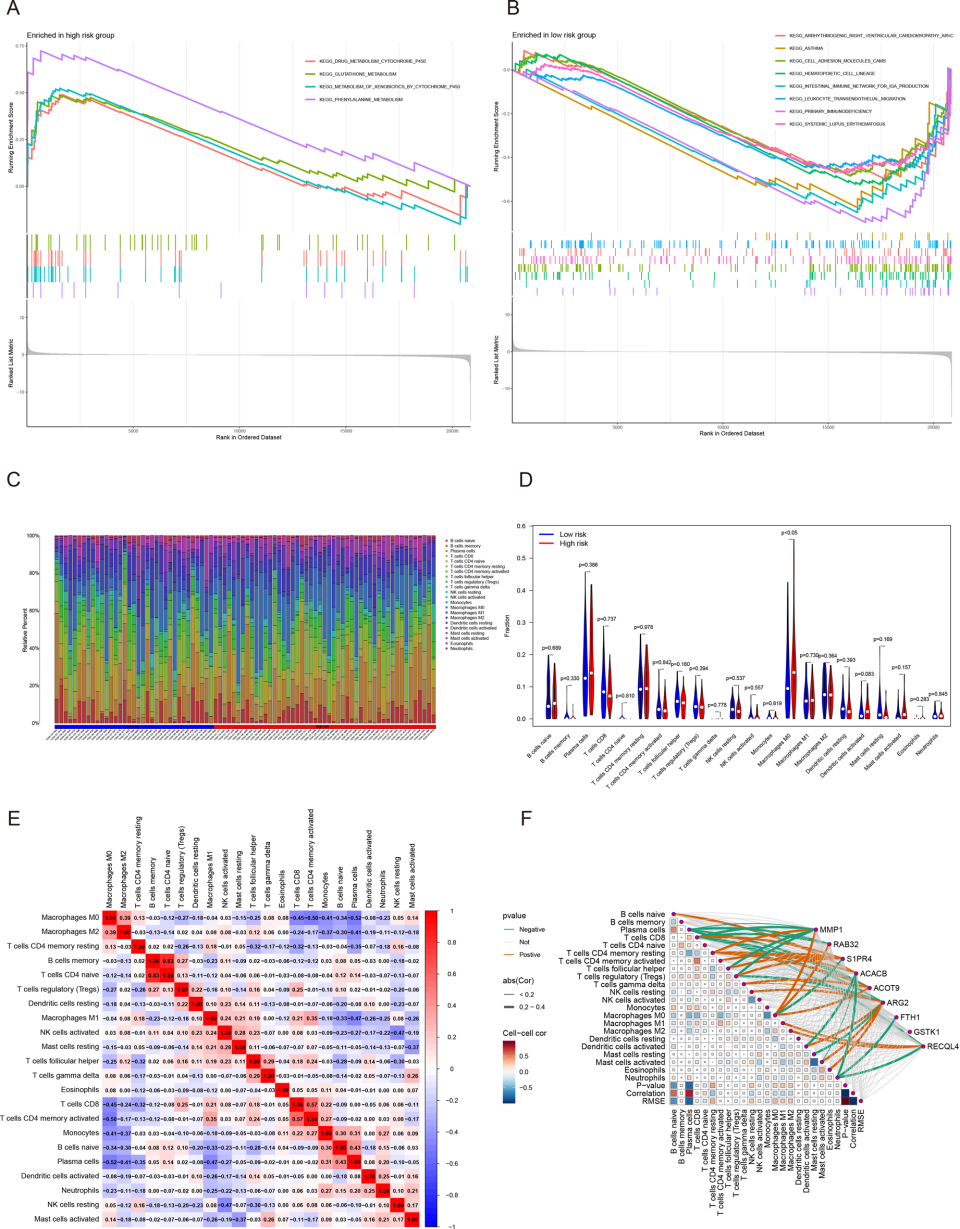
Immune microenvironment landscape of LSCC. (**A-B**) GSEA of KEGG pathways enriched in the high-risk and low-risk groups. (**C**) Distribution of immune cell infiltration among high-risk and low-risk groups based on the MMGS. (**D**) Differential analysis of immune infiltration between the two risk groups. (**E**) Correlation matrix showing interactions and co-infiltration patterns among 22 immune cell types. (**F**) Correlation analysis between immune cell subtypes and the 9 hub genes included in the prognostic model. Abbreviations: LSCC; laryngeal squamous cell carcinoma: MMGS: mitochondrial metabolism-related genes signature; GSEA:Gene set enrichment analysis

**Fig. 6:**
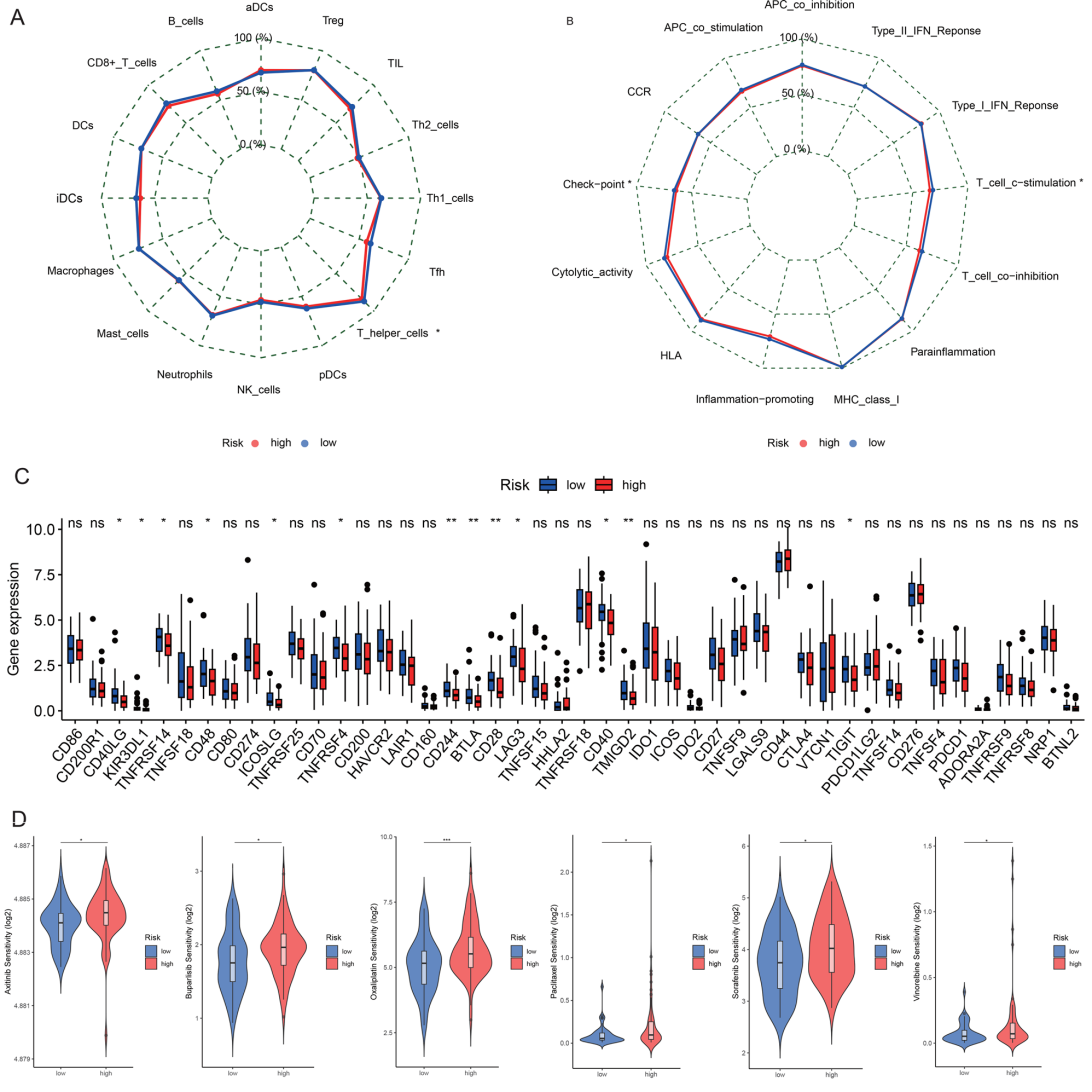
Immune-cell infiltration, immune-pathway activity, immune checkpoint expression, and drug sensitivity differences between the MMGS risk score groups. (**A**) Radar chart illustrating differences in immune-cell infiltration between high- and low- MMGS-risk -score groups, quantified using ssGSEA. (**B**) Radar chart displaying differences in immune-related pathway activity between MMGS -score groups. Pathways include APC co-inhibition, APC co-stimulation, CCR signaling, cytolytic activity, checkpoint pathways, HLA, MHC class I, parainflammation, T cell co-inhibition, T cell co-stimulation, and type I/II IFN responses. (**C**) Comparison of immune checkpoint gene expression between high- and low-risk groups. (**D**) Differential drug sensitivity analysis (estimated IC50 values) for targeted agents and chemotherapeutic drugs. High- and low-risk groups exhibit distinct sensitivities to Axitinib, Buparlisib, oxaliplatin, Paclitaxel, Sorafenib, and Vinorelbine. (Statistical significance is indicated as *p < 0.05, **p < 0.01, ***p < 0.001; ns = not significant). Abbreviations: MMGS: mitochondrial metabolism-related genes signature

To explore the potential therapeutic implications of the MMGS, GSEA was performed using KEGG pathways, which revealed distinct enrichment patterns between the high- and low-risk groups. The high-risk group was primarily enriched in pathways such as drug metabolism—cytochrome P450, glutathione metabolism, metabolism of xenobiotics by cytochrome P450, and phenylalanine metabolism. In contrast, the low-risk group showed significant enrichment in pathways including arrhythmogenic right ventricular cardiomyopathy, asthma, cell adhesion molecules, hematopoietic cell lineage, intestinal immune network for IgA production, leukocyte transendothelial migration, primary immunodeficiency, and systemic lupus erythematosus (Fig. 5A, B). To explore the impact of the MMGS on the tumor immune microenvironment (TIME) in LSCC, we analyzed immune cell infiltration patterns between high-risk and low-risk groups. The distribution of immune cell infiltration, estimated using established algorithms, revealed distinct profiles in high-risk versus low-risk patients based on the MMGS (Fig. 5C). Differential analysis further identified significant differences in the abundance of specific immune cell types between the two risk groups, suggesting that only M0 macrophages were significantly increased in the high-risk group (Fig. 5D). A correlation matrix illustrated that M0 macrophages exhibited significant negative correlations with CD8+ T cells, activated memory CD4+ T cells, monocytes, naïve B cells, plasma cells, and neutrophils (Fig. 5E). Additionally, correlation analysis between immune cell subtypes and the expression of the nine hub genes in the signature revealed a heterogeneous immune–gene interaction pattern. The nine hub genes, including MMP1, RAB32, S1PR4, ACACB, ACOT9, ARG2, FTH1, GSTK1, and RECQL4, showed variable positive or negative associations with multiple immune cell populations. Notably, several hub genes were significantly correlated with CD8+ T cells, plasma cells, macrophage subsets, dendritic cells, mast cells, eosinophils, and neutrophils, whereas naïve CD4+ T cells exhibited relatively weak or nonsignificant associations with the signature. These findings suggest that the MMGS may be closely related to immune-cell infiltration and remodeling of the tumor immune microenvironment (Fig. 5F). Further characterization using ssGSEA confirmed differences in immune-cell infiltration and in the activity of key immune-related pathways between high- and low- MMGS -score groups, as visualized in a radar chart. The results demonstrated significant statistical differences between the high-risk and low-risk groups in the activity of T helper cells, T cell co-stimulation, and checkpoint pathways (Fig. 6A, B). Examination of immune checkpoint gene expression showed significant differences between high- and low-risk groups for several key checkpoints, suggesting that CD200R1, CD40LG, TNFRSF14, CD48, ICOSLG, TNFRSF4, CD244, BTLA, CD28, LAG3, CD40, and TMIGD2 were significantly downregulated in the high-risk group compared to the low-risk group (Fig. 6C). Finally, differential drug sensitivity analysis, based on estimated half-maximal inhibitory concentration (IC50) values, identified distinct sensitivities to various targeted agents and chemotherapeutic drugs between the high- and low-risk groups. Notably, the high-risk group demonstrated altered responses to drugs such as Axitinib, Buparlisib, Oxaliplatin, Paclitaxel, Sorafenib, and Vinorelbine, providing insights into potential personalized therapeutic strategies guided by the MMGS stratification (Fig. 6D).

### The expression levels of MMGs in LSCC patients

**Fig. 7:**
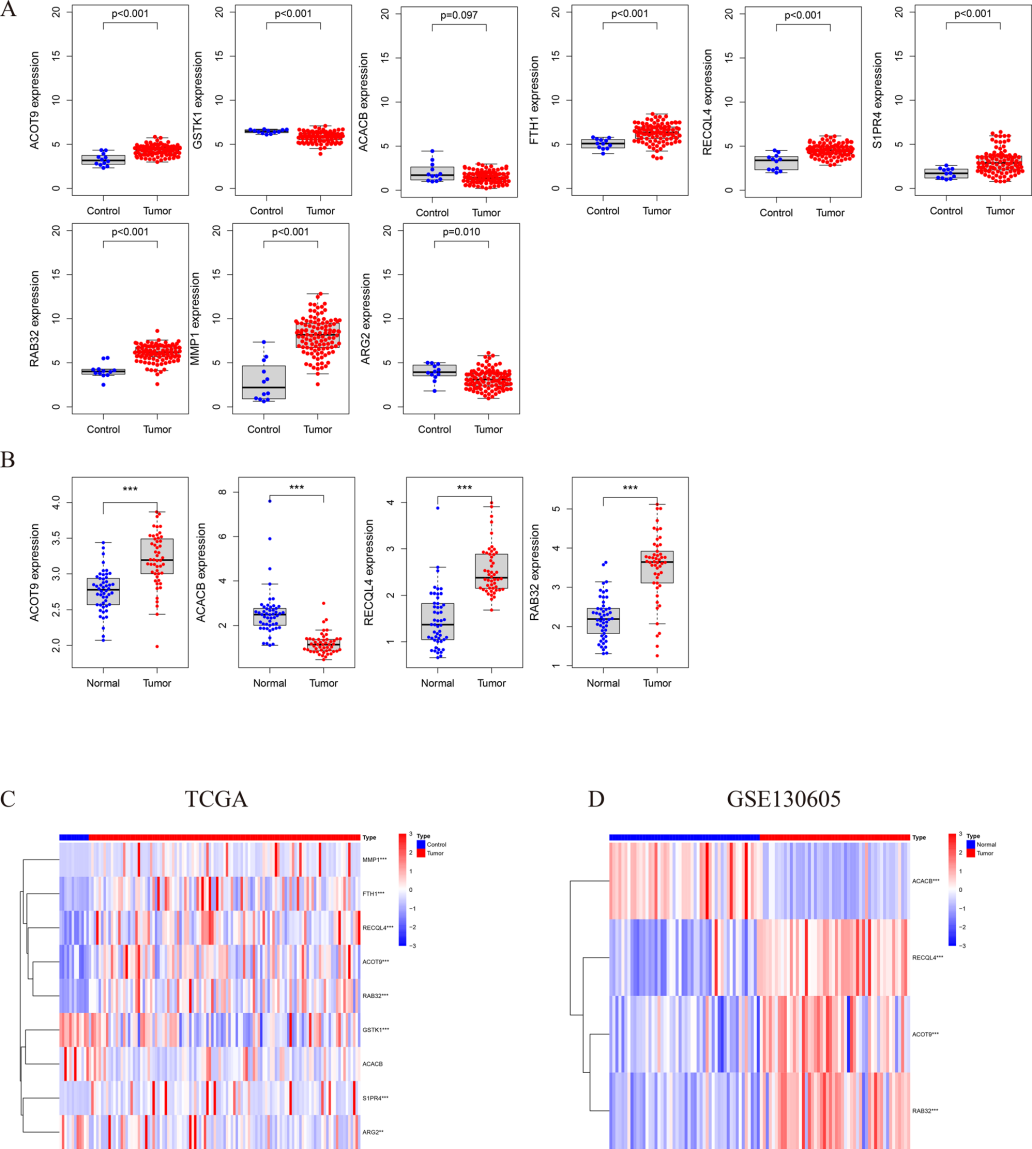
Expression patterns of the nine MMGs in LSCC across TCGA and GEO datasets. (**A, C**) Differential expression of the nine MMGs (FTH1, ACOT9, MMP1, RAB32, S1PR4, RECQL4, ACACB, GSTK1, and ARG2) between control and tumor tissues in the TCGA-LSCC cohort. (**B, D**) Validation of the expression patterns of these nine MMGs using the GEO dataset GSE130605. Abbreviations: MMGs: mitochondrial metabolism-related genes; LSCC; laryngeal squamous cell carcinoma

We analyzed the expression levels of the 9 MMGs in normal tissues and LSCC samples. The results showed that FTH1, ACOT9, MMP1, RAB32, S1PR4, and RECQL4 were significantly upregulated in LSCC, whereas ACACB, GSTK1, and ARG2 were significantly downregulated (Fig. 7A, C). Furthermore, the expression patterns of these 9 MMGs were validated using the GEO dataset GSE130605. The validation results confirmed that ACOT9, RAB32, and RECQL4 were markedly upregulated in LSCC patients, while ACACB was significantly downregulated (Fig. 7B, D).

**Fig. 8:**
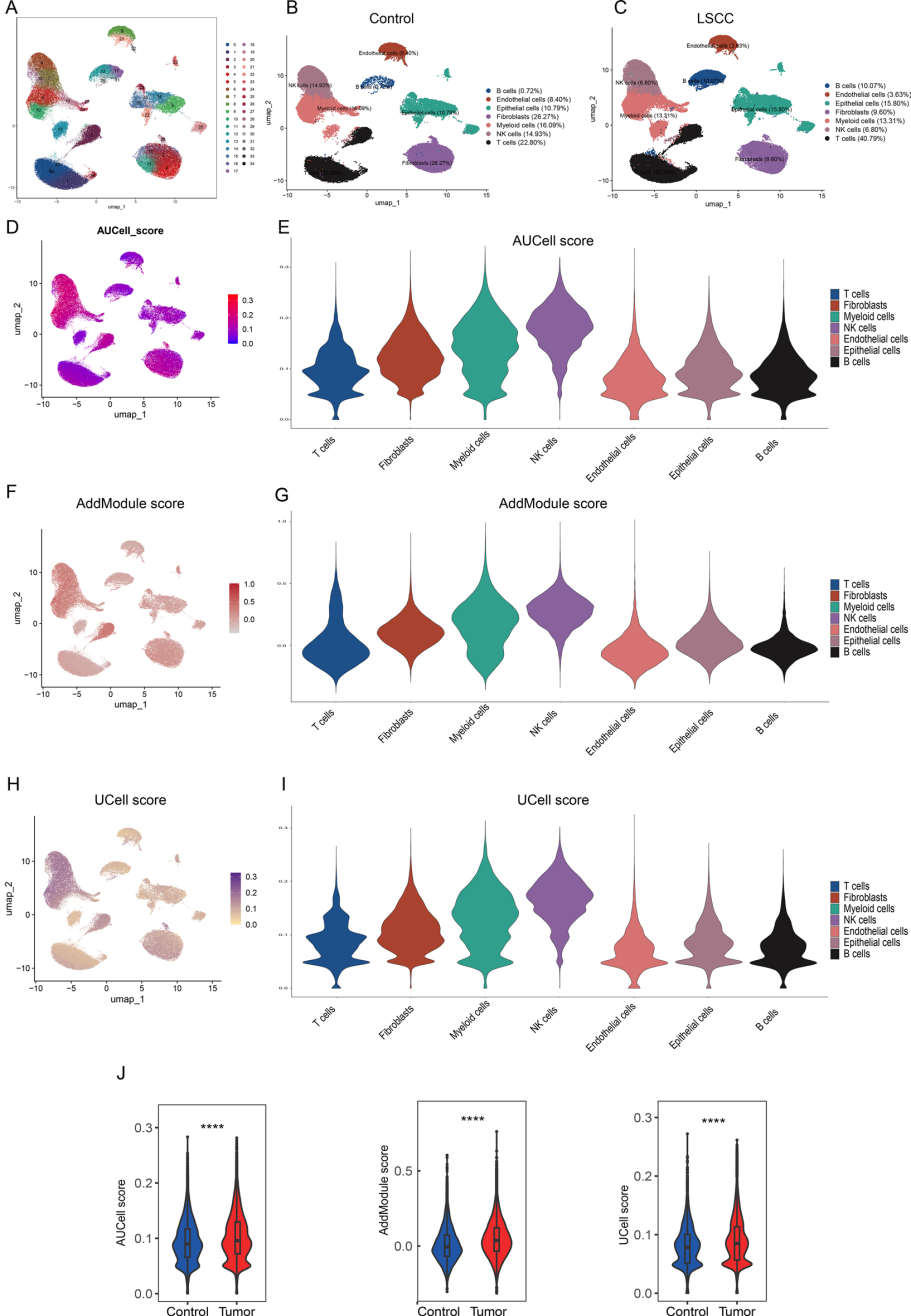
Single-cell analysis of MMGs in LSCC (GSE290927 dataset). (**A**) UMAP clustering of all cells from GSE290927, showing 35 transcriptionally distinct clusters. (**B**) Cell-type annotation of the control samples. (**C**) Cell-type annotation of the LSCC samples. UMAP plots displaying the enrichment scores of the 9 MMGs calculated by AUCell (**D**), AddModule (**F**), and UCell (**H**) algorithms, respectively; higher scores are shown in red. (**E, G, I**) Violin plots illustrating the distribution of AUCell (**E**), AddModule (**G**), and UCell (**I**) scores across the seven major cell types (T cells, fibroblasts, myeloid cells, NK cells, endothelial cells, epithelial cells, and B cells). (**J**) Violin plots comparing the global AUCell, AddModule, and UCell scores between control and LSCC groups. (Statistical significance is indicated as *p < 0.05, **p < 0.01, **p < 0.001.). Abbreviations: MMGs: mitochondrial metabolism-related genes; LSCC; laryngeal squamous cell carcinoma

To further elucidate the cellular heterogeneity and expression patterns of the 9 MMGs in LSCC at single-cell resolution, we analyzed the publicly available single-cell RNA-seq dataset (GSE290927). Uniform Manifold Approximation and Projection (UMAP) dimensionality reduction revealed 35 transcriptionally distinct cell clusters across all samples (Fig. 8A). Separate cell-type annotation of control (vocal cord polyp) and LSCC samples identified the major cell populations, including T cells, fibroblasts, myeloid cells, NK cells, endothelial cells, epithelial cells, and B cells. Cell identities were confirmed by UMAP visualization with established marker genes (Supplementary Fig. S1). Notably, the two groups displayed distinct cellular composition patterns (Fig. 8B, C). We next calculated module scores for the 9 MMGs in each cell using three complementary algorithms: AUCell, AddModuleScore, and UCell. UMAP projections of these enrichment scores demonstrated spatially distinct patterns of MRGs activity, with higher scores (indicated in red) prominently enriched in certain cell populations, particularly in LSCC samples (Fig. 8D, F, H). Violin plots further illustrated the distribution of AUCell, AddModule, and UCell scores across the seven major cell types. Notably, immune cell subsets (myeloid cells and NK cells) exhibited relatively higher MMGS scores compared to other cell types, suggesting cell-type-specific mitochondrial reprogramming in the tumor microenvironment (Fig. 8E, G, I). Global comparison of the module scores between control and LSCC groups revealed significantly elevated AUCell, AddModule, and UCell scores in LSCC samples compared to normal tissues (Fig. 8J).

### Interpretability analysis of the MMGs and validation of key gene ACOT9

**Fig. 9:**
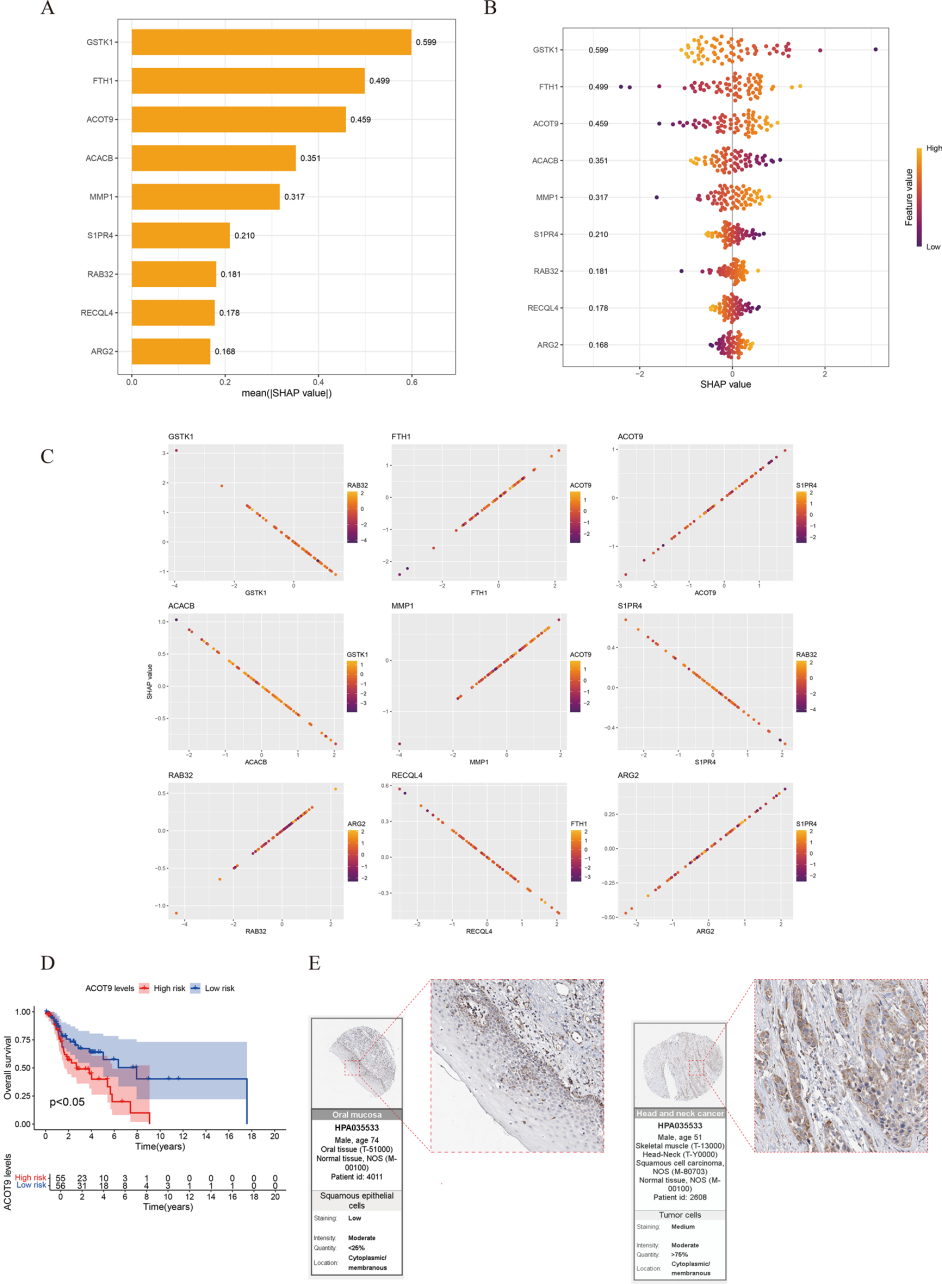
SHAP interpretation of the MMGs and ACOT9 validation in LSCC. (**A**) SHAP feature importance ranking the contributions of model variables to the predicted MMGS risk score. (**B**) SHAP summary plot showing the distribution of SHAP values across LSCC samples, where dot colors reflect gene expression levels and positions indicate their impact on risk prediction. (**C**) SHAP dependence plot demonstrating how changes in key MMGs influence individual patient risk estimates. (**D**) Kaplan–Meier survival analysis of ACOT9 in the TCGA LSCC cohort. (**E**) IHC analysis of ACOT9 protein expression in the HPA database. Abbreviations: SHAP: shapley additive Explanations; MMGs: mitochondrial metabolism-related genes; LSCC; laryngeal squamous cell carcinoma; MMGS: mitochondrial metabolism-related genes signature; IHC:Immunohistochemistry; HPA: human protein Atlas

To further interpret feature importance in the optimal machine-learning model, SHAP analysis was performed on the candidate hub genes. As shown in Fig. 9A, GSTK1 exhibited the highest contribution to model output, followed by FTH1, ACOT9, ACACB, MMP1, S1PR4, RAB32, RECQL4, and ARG2, indicating that these genes represented the most influential features in the prognostic model. The SHAP summary plot further demonstrated the direction and magnitude of each gene’s contribution to model prediction (Fig. 9B). In general, higher expression levels of FTH1, ACOT9, MMP1, RAB32, and ARG2 were associated with increased SHAP values, suggesting positive contributions to the predicted outcome, whereas higher expression levels of GSTK1, ACACB, S1PR4, and RECQL4 tended to correspond to lower SHAP values. Consistently, the SHAP dependence plots showed largely monotonic relationships between gene expression and SHAP values, with FTH1, ACOT9, MMP1, RAB32, and ARG2 displaying positive trends, while GSTK1, ACACB, S1PR4, and RECQL4 exhibited negative trends (Fig. 9C). Collectively, these findings identified GSTK1, FTH1, ACOT9, ACACB, and MMP1 as the top-ranked contributors to model prediction, highlighting their potential importance in the MMGS. Among the identified prognostic genes, ACOT9 was selected for further validation because it not only ranked among the top weighted genes in the SHAP analysis but also showed significant prognostic relevance in the TCGA LSCC cohort (HR = 1.959, 95% CI: 1.142–3.362, *p* = 0.015; Fig. 1G). In addition, ACOT9 was consistently upregulated in LSCC tissues in both the TCGA and GSE130605 cohorts (Fig. 7A, B). These findings indicated that ACOT9 was not only associated with poor prognosis but also contributed substantially to the predictive model. Therefore, ACOT9 was chosen as the key candidate for subsequent experimental validation. Kaplan–Meier analysis in the TCGA-LSCC cohort revealed significantly poorer overall survival in patients with high ACOT9 expression (log-rank *p* < 0.05) (Fig. 9D). Immunohistochemistry data from the Human Protein Atlas confirmed markedly elevated ACOT9 protein expression in LSCC tumor tissues compared to normal laryngeal epithelium (Fig. 9E).

### Single-cell analysis of ACOT9 expression, epithelial subtype heterogeneity, and functional implications in LSCC

**Fig. 10:**
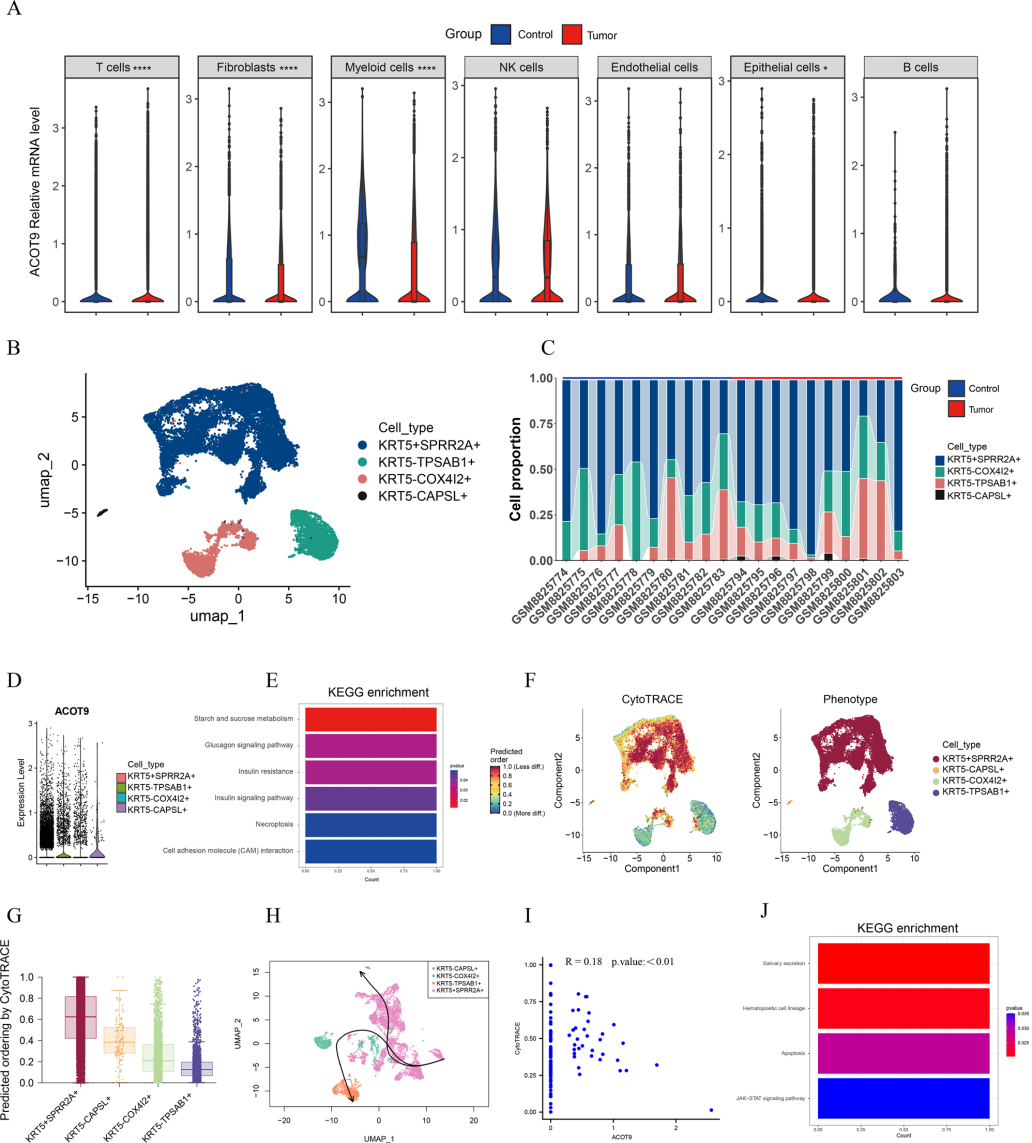
Single-cell analysis of ACOT9 expression in epithelial-cell subtypes functional enrichment in GSE290927. (**A**) Violin plots showing ACOT9 expression across major cell types (T cells, fibroblasts, myeloid cells, NK cells, endothelial cells, epithelial cells, and B cells) in the control and LSCC groups of the GSE290927 dataset. (**B**) UMAP visualization of epithelial-cell subclusters, identifying four transcriptionally distinct epithelial subtypes: KRT5+SPRR2A+, KRT5–TPSAB1+, KRT5–COX4I2+, and KRT5-CAPSL+. (**C**) Bar plot demonstrating the proportional distribution of the four epithelial-cell subtypes between control and LSCC groups, showing marked subtype compositional changes in tumor samples. (**D**) Violin plots displaying ACOT9 expression across the four epithelial-cell subtypes. (**E**) KEGG pathway enrichment analysis of epithelial cells (tumor vs. control). (**F**) CytoTRACE analysis showing predicted cellular differentiation states of epithelial-cell subtypes. (**G**) Boxplots comparing CytoTRACE-inferred stemness among the four epithelial-cell subtypes. (**H**) Pseudotime trajectory analysis of epithelial-cell subtypes by Slingshot. (**I**) Scatter plot demonstrating a significant positive correlation between CytoTRACE scores and ACOT9 expression in epithelial-cell (*R* = 0.18,*p* < 0.01). (**J**) KEGG enrichment analysis of ACOT9-high epithelial cells. Abbreviations: LSCC; laryngeal squamous cell carcinoma

To investigate the cell-type-specific expression and functional role of ACOT9 in LSCC, we analyzed its expression patterns in the GSE290927 single-cell RNA-seq dataset. Violin plots revealed that ACOT9 expression was markedly higher in epithelial cells compared to other major cell types in both control and LSCC groups, with a pronounced upregulation in LSCC epithelial cells (Fig. 10A). GO and KEGG enrichment analyses were conducted for each single-cell subpopulation (Supplementary Figures S2–S8). Among these, LSCC epithelial cells were characterized by prominent squamous epithelial differentiation features, together with enhanced inflammatory signaling and extracellular matrix remodeling, indicating their active involvement in tumor progression and microenvironmental interactions. Given the prominent biological features observed in epithelial cells, we further performed subclustering analysis of this population. This analysis identified four transcriptionally distinct epithelial subtypes based on their marker gene expression profiles: KRT5+SPRR2A+, KRT5−TPSAB1+, KRT5−COX4I2+, and KRT5−CAPSL+ (Fig. 10B). Bar plot analysis of subtype proportions demonstrated significant compositional shifts in LSCC samples compared to control tissues, with altered representation of these epithelial subtypes in the tumor microenvironment (Fig. 10C). Violin plots further showed differential ACOT9 expression across these four epithelial subtypes, with the highest levels observed in specific tumor-enriched subtypes (Fig. 10D). KEGG pathway enrichment analysis of epithelial cells highlighted pathways associated with tumor progression, metabolic reprogramming, and epithelial-mesenchymal transition in LSCC epithelial cells (Fig. 10E). CytoTRACE analysis predicted cellular differentiation states, revealing varying degrees of stemness among the four epithelial subtypes (Fig. 10F). Pseudotime trajectory inference using Slingshot delineated developmental trajectories of epithelial-cell subtypes, suggesting dynamic transitions in the tumor context (Fig. 10G). Notably, a significant positive correlation was observed between CytoTRACE-inferred stemness scores and ACOT9 expression levels in epithelial cells (*R* = 0.18, *p* < 0.01), indicating that higher ACOT9 expression is associated with increased stem-like properties (Fig. 10H). KEGG enrichment analysis of ACOT9-high epithelial cells further identified pathways related to Mitochondrial metabolism, oxidative stress response, and oncogenic signaling, supporting a potential role for ACOT9 in promoting stemness and malignant progression in LSCC epithelial subpopulations (Fig. 10I).

### Experimental validation of ACOT9 upregulation and oncogenic role in LSCC progression

**Fig. 11:**
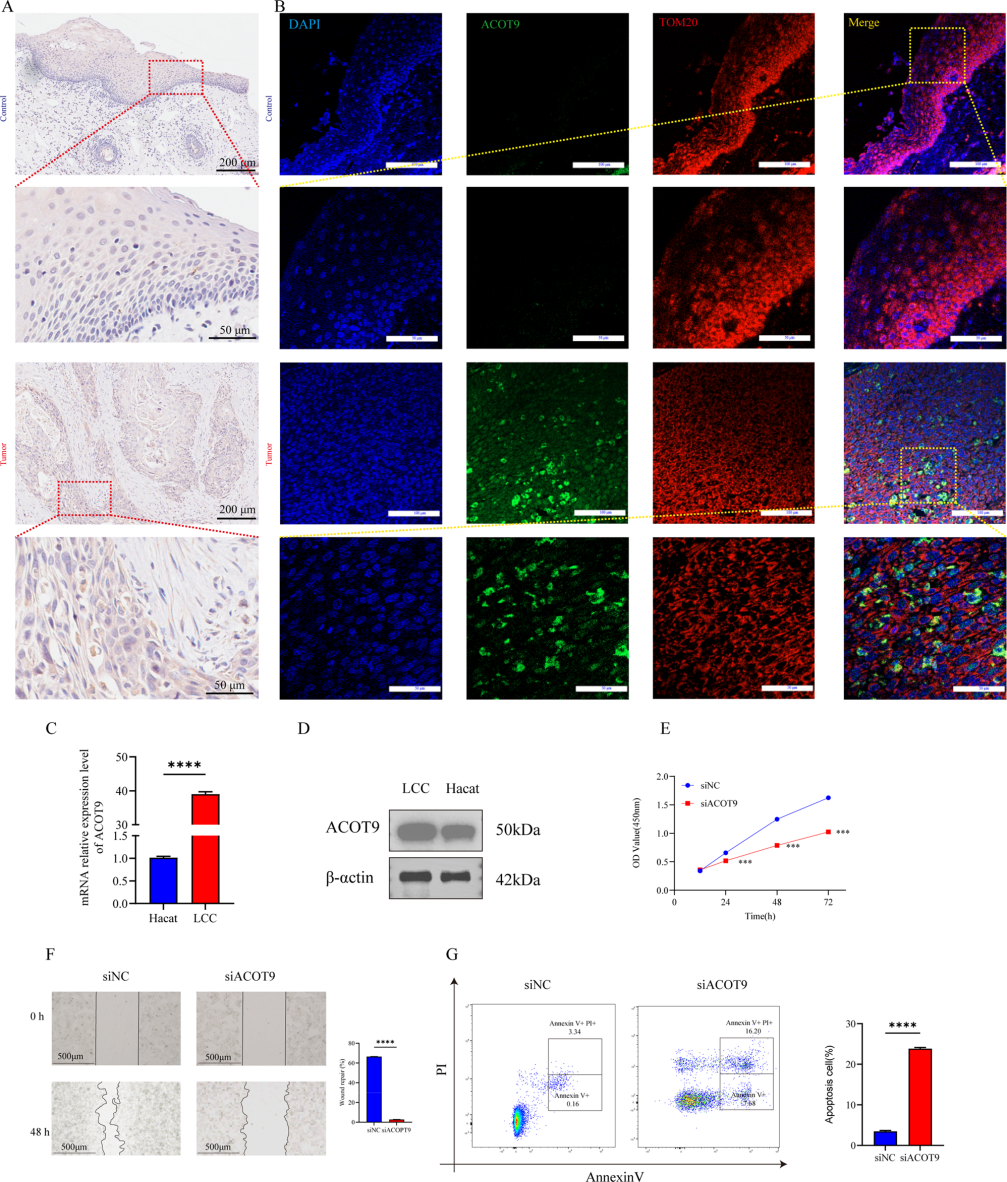
ACOT9 is significantly upregulated in LSCC and promotes malignant phenotypes in laryngeal cancer cells. (**A**) Representative immunohistochemistry images of ACOT9 in normal laryngeal tissues and LSCC tissue samples. (**B**) Representative co-immunofluorescence staining of ACOT9 (green) and the mitochondrial marker TOM20 (red) in normal and LSCC groups. (**C**) Real-time quantitative PCR analysis of ACOT9 mRNA expression levels in HaCaT cells and LCC cells. (**D**) Western blotting analysis of ACOT9 protein expression levels in HaCaT cells and LCC cells. (**E**) CCK-8 assay showing the viability of LCC cells at different time points following ACOT9 knockdown. (**F**) Wound-healing (scratch) assay assessing the migration ability of LCC cells at 48 hours following ACOT9 knockdown. (**G**) Flow cytometric analysis of apoptosis rates in LCC cells following ACOT9 knockdown. Abbreviations: LSCC; laryngeal squamous cell carcinoma

To validate the clinical relevance of ACOT9 identified through bioinformatics analyses, we first examined its expression in clinical LSCC specimens and laryngeal cancer cell lines. Immunohistochemistry (IHC) showed that ACOT9 protein expression was markedly elevated in LSCC tissues compared with adjacent normal laryngeal epithelium (Fig. 11A). Consistently, co-immunofluorescence staining demonstrated prominent co-localization of ACOT9 with the mitochondrial marker TOM20 in LSCC tissues, whereas only weak staining was observed in normal tissues, supporting mitochondrial enrichment of ACOT9 in laryngeal cancer cells (Fig. 11B). In vitro, both quantitative real-time PCR and Western blotting confirmed that ACOT9 mRNA and protein levels were significantly higher in LCC cells than in HaCaT cells (Fig. 11C, D), in agreement with the expression pattern observed in public datasets.

To further investigate the functional role of ACOT9 in LSCC progression, we performed knockdown experiments in LCC cells. ACOT9 silencing significantly reduced cell viability in a time-dependent manner, as determined by the CCK-8 assay (Fig. 11E). In addition, wound-healing assays showed that ACOT9 knockdown markedly impaired the migratory capacity of LCC cells at 48 h (Fig. 11F). Flow cytometric analysis further revealed a significant increase in apoptotic cells following ACOT9 depletion (Fig. 11G). Collectively, these findings indicate that ACOT9 is significantly upregulated in LSCC and promotes malignant phenotypes by enhancing tumor cell viability and migration while suppressing apoptosis.

### Virtual knockout analysis links ACOT9 to a LAMC2/KRT17-centered epithelial remodeling network and supports a role for mitochondrial ROS in LSCC malignancy

**Fig. 12:**
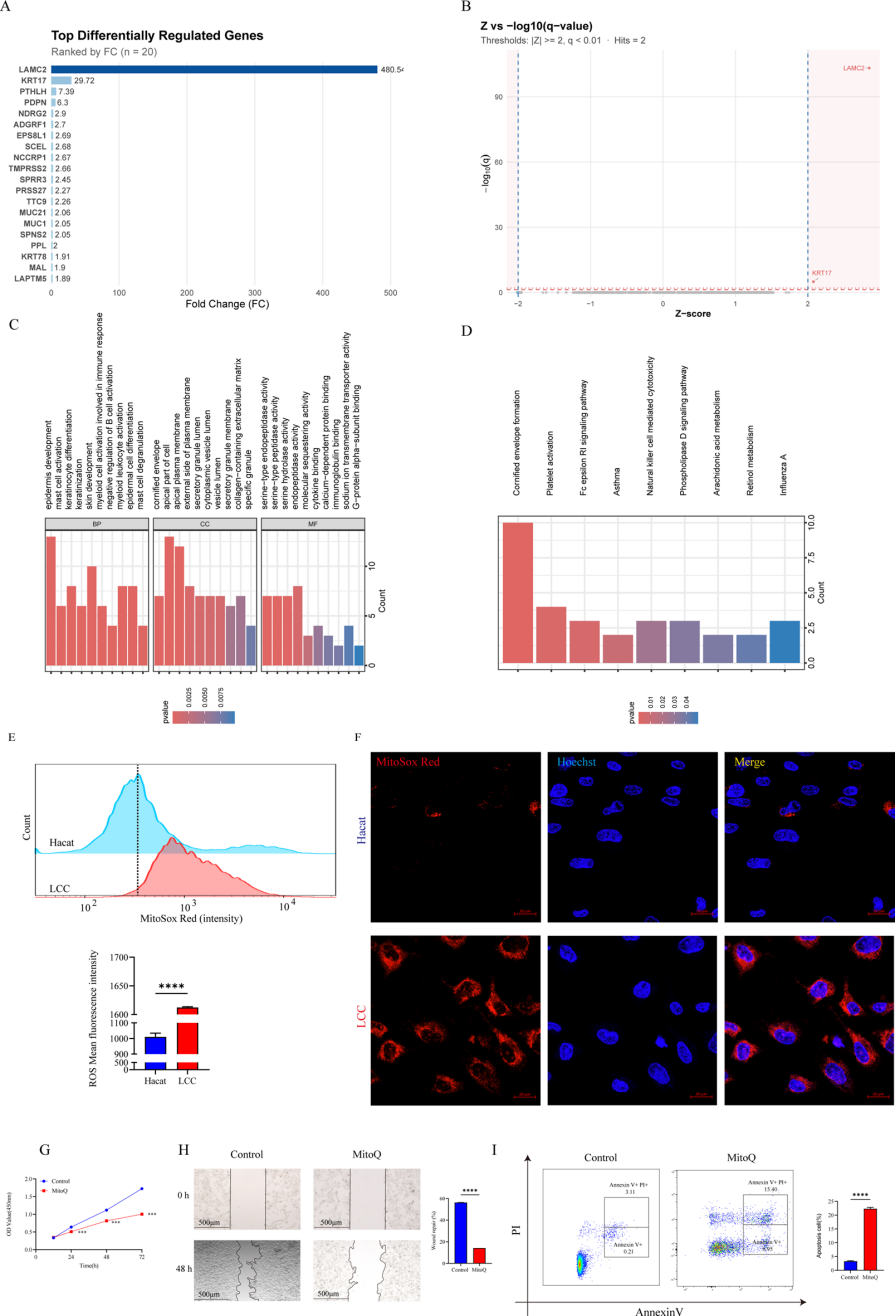
ACOT9 virtual knockout reveals a LAMC2/KRT17-centered epithelial remodeling program, and mitochondrial ROS promotes malignant phenotypes in LCC cells. (**A**) Top differentially regulated genes ranked by FC after ACOT9 virtual knockout in epithelial cells. (**B**) Z score versus −log10(q value) plot showing LAMC2 and KRT17 as the two significant perturbed genes. (**C**) GO enrichment analysis of the top 100 perturbed genes ranked by Z score, showing enrichment in epithelial development/remodeling, keratinization, immune activation, and secretory/vesicle-associated processes. (**D**) KEGG pathway enrichment analysis of the top 100 perturbed genes ranked by Z score, indicating enrichment in inflammatory/immune-related signaling, lipid inflammatory signaling, and epithelial barrier/remodeling pathways. (**E**) Flow cytometric analysis of mitochondrial ROS levels in HaCaT cells and LCC cells. (**F**) Immunofluorescence detection of mitochondrial reactive oxygen species (ROS) levels in HaCaT cells and LCC cells. (**G**) CCK-8 assay showing the viability of LCC cells at different time points with or without MitoQ treatment (a mitochondrial ROS scavenger). (**H**) Wound-healing (scratch) assay assessing the migration ability of LCC cells at 48 hours with or without MitoQ treatment (a mitochondrial ROS scavenger). (**I**) Flow cytometric analysis of apoptosis rates in LCC cells with or without MitoQ treatment (a mitochondrial ROS scavenger)

To further explore the downstream regulatory consequences of ACOT9 loss, we performed scTenifoldKnk-based virtual knockout analysis in epithelial cells. Ranking perturbed genes by fold change identified a subset of strongly regulated candidates after in silico deletion of ACOT9 (Fig. 12A). Notably, the Z score versus −log10 (q value) plot showed that, under the thresholds of |Z| ≥2 and q < 0.01, LAMC2 and KRT17 emerged as the two most significant perturbed genes (Fig. 12B). Functional enrichment analysis of the top 100 perturbed genes ranked by Z score revealed significant enrichment in epithelial development/remodeling, keratinization, immune activation, and secretory/vesicle-associated processes in GO analysis (Fig. 12C), whereas KEGG analysis highlighted inflammatory/immune-related signaling, lipid inflammatory signaling, and epithelial barrier/remodeling pathways (Fig. 12D). These findings suggest that ACOT9 deficiency preferentially disrupts a downstream epithelial stress-remodeling network centered on LAMC2/KRT17.

Given the mitochondrial localization of ACOT9, we next assessed whether oxidative stress might represent a mechanistic link between ACOT9 and its downstream malignant network. Flow cytometric analysis demonstrated that mitochondrial ROS levels were significantly higher in LCC cells than in HaCaT cells (Fig. 12E), which was further confirmed by MitoSOX-based immunofluorescence staining (Fig. 12F). To determine whether elevated mitochondrial ROS contributes functionally to the malignant phenotype, LCC cells were treated with MitoQ, a mitochondria-targeted ROS scavenger. MitoQ treatment significantly reduced cell viability over time (Fig. 12G), suppressed cell migration in wound-healing assays at 48 h (Fig. 12H), and increased apoptosis rates in LCC cells (Fig. 12I). Taken together, these results indicate that mitochondrial ROS is functionally involved in maintaining malignant phenotypes in LCC cells, and, in combination with the virtual knockout findings, support a model in which ACOT9 contributes to LSCC progression through a downstream epithelial remodeling program potentially linked to mitochondrial redox dysregulation.

## Discussion

In the present study, we comprehensively explored the role of MMGs in LSCC through integrated bioinformatics, machine learning, immune microenvironment analysis, and single-cell transcriptomics. Our findings establish a novel 9-hub-gene signature as a robust, independent prognostic tool with excellent discriminatory performance (C-index > 0.7388) across training, test, and external validation cohorts, highlighting the central contribution of mitochondrial metabolic reprogramming to LSCC progression, immune evasion, and therapeutic response.

Mitochondrial dysfunction is increasingly recognized as a hallmark of cancer metabolism [31–34]. By identifying MMGs and applying WGCNA, we uncovered co-expression modules (particularly MEgreen and MEgrey) that strongly correlate with LSCC status, consistent with recent studies demonstrating that MMGs drive tumorigenesis and serve as prognostic biomarkers in LSCC and HNSCC [35, 36]. The strong negative correlation of the MEgreen module and positive correlation of the MEgrey module with tumor trait align with the concept that suppression of protective mitochondrial functions and activation of oncogenic mitochondrial pathways promote malignant transformation [37, 38].

The optimal MMGs, identified as univariate Cox candidates from the intersection of MMGs and key module genes, outperformed many previously reported signatures [39, 40]. Its high long-term predictive accuracy (highest AUC at 5 years) and independent prognostic value in multivariate Cox analysis (beyond age, grade, and T/N stage) support its clinical translational potential, as evidenced by the well-calibrated nomogram. Spearman correlation and SHAP interpretability analyses further revealed distinct gene-specific contributions to model-predicted risk. FTH1, ACOT9, MMP1, RAB32, and ARG2 were positively associated with the predicted risk score, suggesting risk-promoting effects, whereas GSTK1, ACACB, S1PR4, and RECQL4 showed opposite trends and tended to contribute negatively to risk prediction. These SHAP-derived patterns were broadly consistent with the differential expression profiles observed in TCGA and GSE130605, as well as the protein-level evidence from the HPA database. Among these genes, ACOT9, a fatty acid metabolism-related enzyme, emerged as one of the key high-risk candidates, showing consistent upregulation, association with poorer survival, and epithelial-specific enrichment. These findings further extend previous fatty acid metabolism-related prognostic signatures in LSCC in which ACOT9 was also identified as a prognostic factor [41].

Immune microenvironment analyses revealed that the MMGS stratifies patients into distinct tumor immune microenvironments (TIME). The high-risk group exhibited increased M0 macrophage infiltration with negative correlations to anti-tumor effector cells (CD8+ T cells, activated CD4+ memory T cells, etc.), alongside positive associations between M0 macrophages and multiple high-risk MMGs (including ACOT9). This pro-tumorigenic macrophage polarization, coupled with downregulated T cell co-stimulation, elevated checkpoint pathway activity, and reduced expression of co-stimulatory molecules (CD28, ICOSLG, TNFRSF4, etc.), indicates an immunosuppressive TIME that likely contributes to worse prognosis [42, 43]. These observations are in line with single-cell studies showing altered immune cell composition and epithelial-immune crosstalk in LSCC [44, 45].

Pathway enrichment further supports metabolic-immune crosstalk: high-risk tumors were enriched in drug metabolism (cytochrome P450, glutathione) and xenobiotic pathways, potentially explaining altered drug sensitivities (to Axitinib, Sorafenib, Paclitaxel), while low-risk tumors showed enrichment in immune activation pathways (leukocyte transendothelial migration, IgA production). Differential drug response predictions suggest that MMGS-based stratification could guide personalized therapy, such as combining mitochondrial-targeted agents with immune checkpoint inhibitors in high-risk patients.

Single-cell resolution analysis (GSE290927) provided deeper mechanistic insights. The MMGS scores were significantly elevated in LSCC versus normal tissues, with prominent enrichment in epithelial and myeloid/NK cells, confirming cell-type-specific mitochondrial reprogramming. Focused on ACOT9—the gene with consistent prognostic and functional prominence—we found its highest expression in malignant epithelial subtypes, positive correlation with CytoTRACE-inferred stemness, and enrichment of ACOT9-high epithelial cells in Mitochondrial metabolism, oxidative stress, and oncogenic pathways. In addition, scTenifoldKnk-based virtual knockout of ACOT9 in epithelial cells identified LAMC2 and KRT17 as the two most significantly perturbed genes, while GO and KEGG analyses of the top perturbed genes highlighted epithelial remodeling, keratinization, extracellular matrix organization, inflammatory/immune signaling, and lipid inflammatory pathways. These findings suggest that ACOT9 loss preferentially disrupts a downstream epithelial stress-remodeling network centered on LAMC2/KRT17, thereby extending recent single-cell atlases of LSCC epithelial heterogeneity and stemness [46, 47].

Experimental validation provided direct evidence of ACOT9‘s oncogenic function. IHC and co-IF confirmed mitochondrial-localized ACOT9 overexpression in LSCC tissues and LCC cells. In addition, mitochondrial ROS levels were significantly elevated in LCC cells compared with HaCaT cells, and pharmacological scavenging of mitochondrial ROS by MitoQ suppressed viability and migration while promoting apoptosis, supporting a functional role for mitochondrial ROS in maintaining malignant phenotypes. This observation is consistent with previous evidence that pharmacological modulation of mitochondrial dysfunction, particularly through mitochondria-targeted antioxidants, may reduce ROS accumulation and influence mitochondrial quality control, biogenesis, and inflammation-related signaling [48]. Consistently, ACOT9 knockdown decreased viability, inhibited migration, and increased apoptosis in LCC cells. However, these findings should be interpreted as supporting an association between ACOT9, mitochondrial ROS accumulation, and malignant phenotypes, rather than definitive evidence that ACOT9 directly induces ROS generation. Given that ACOT9 is a fatty acid metabolism-related mitochondrial enzyme, ACOT9 may contribute to mitochondrial redox dysregulation by affecting fatty acid metabolic reprogramming, mitochondrial acyl-CoA homeostasis, oxidative phosphorylation, mitochondrial respiratory activity, and oxidative stress signaling. Together with the virtual knockout findings, these results support a model in which ACOT9 promotes LSCC aggressiveness and is potentially linked to a mitochondrial redox-dependent epithelial remodeling program centered on LAMC2/KRT17. These findings extend prior reports linking ACOT9 to fatty acid metabolism and oncogenic progression [41], and align with studies showing that mitochondrial ROS signaling [49, 50], LAMC2-mediated ER-mitochondria adaptation [51], and KRT17-associated stemness/chemoresistance contribute to cancer progression and therapeutic resistance [52].

Our study has several strengths, including multi-algorithm machine learning validation, external cohorts, SHAP interpretability, and multi-omics (bulk + single-cell) integration. However, limitations include reliance on retrospective cohorts without prospective validation, potential confounding by batch effects in public datasets, lack of in vivo validation, and the need for broader functional studies. In particular, although our data support an association between ACOT9, mitochondrial ROS, and malignant phenotypes, the direct causal mechanism by which ACOT9 regulates ROS accumulation remains to be fully elucidated. Future studies incorporating xenograft models using ACOT9-knockdown LCC cells, metabolic flux analysis, mitochondrial respiration assays, and rescue experiments are warranted to further assess tumor growth, apoptosis, mitochondrial ROS-related markers, and LAMC2/KRT17 expression in vivo. Future work should also validate the signature in multi-center cohorts, investigate ACOT9-ferroptosis/stemness crosstalk, and test mitochondrial-targeted agents in combination with immunotherapy.

## Conclusions

In conclusion, our integrated multi-omics and functional study demonstrates that mitochondrial reprogramming, particularly involving ACOT9 and mitochondrial redox dysregulation, plays a pivotal role in LSCC progression, immune suppression, and prognosis. Our results further suggest that ACOT9 loss disrupts a downstream epithelial stress-remodeling network centered on LAMC2/KRT17, while mitochondrial ROS contributes functionally to the maintenance of malignant phenotypes in LCC cells. The MMGS offers a reliable and interpretable tool for patient stratification and personalized therapy guidance, while targeting ACOT9 or mitochondrial ROS-related pathways may represent novel therapeutic strategies.

## Electronic supplementary material

Below is the link to the electronic supplementary material.

Supplementary material 1

Supplementary material 2

Supplementary material 3

Supplementary material 4

Supplementary material 5

Supplementary material 6

Supplementary material 7

Supplementary material 8

Supplementary material 9

## Data Availability

The datasets analyzed in the current study are publicly available from The Cancer Genome Atlas (TCGA) database at the GDC Data Portal and the Gene Expression Omnibus (GEO) database. Additional data generated or analyzed during this study are available from the corresponding author upon reasonable request.

## Abbreviations

LSCC: Laryngeal squamous cell carcinoma
MMGs: Mitochondrial metabolism-related genes
MMGS: Mitochondrial metabolism-related genes signature
ROS: Reactive oxygen species
TCGA: The Cancer Genome Atlas
GEO: Gene Expression Omnibus
TIME: Tumor immune microenvironment
WGCNA: Weighted gene co-expression network analysis
GSEA: Gene set enrichment analysis
GO: Gene Ontology
KEGG: Kyoto Encyclopedia of Genes and Genomes
IHC: Immunohistochemistry
IF: Immunofluorescence
