# Supplementary figures and images for: ACOT9, a mitochondrial metabolism-related gene, promotes ROS-associated epithelial remodeling in laryngeal squamous cell carcinoma
Source: J Transl Med. 2026 Jun 24;24:917. doi: 10.1186/s12967-026-08470-x (PMC13374139; doi:10.1186/s12967-026-08470-x)

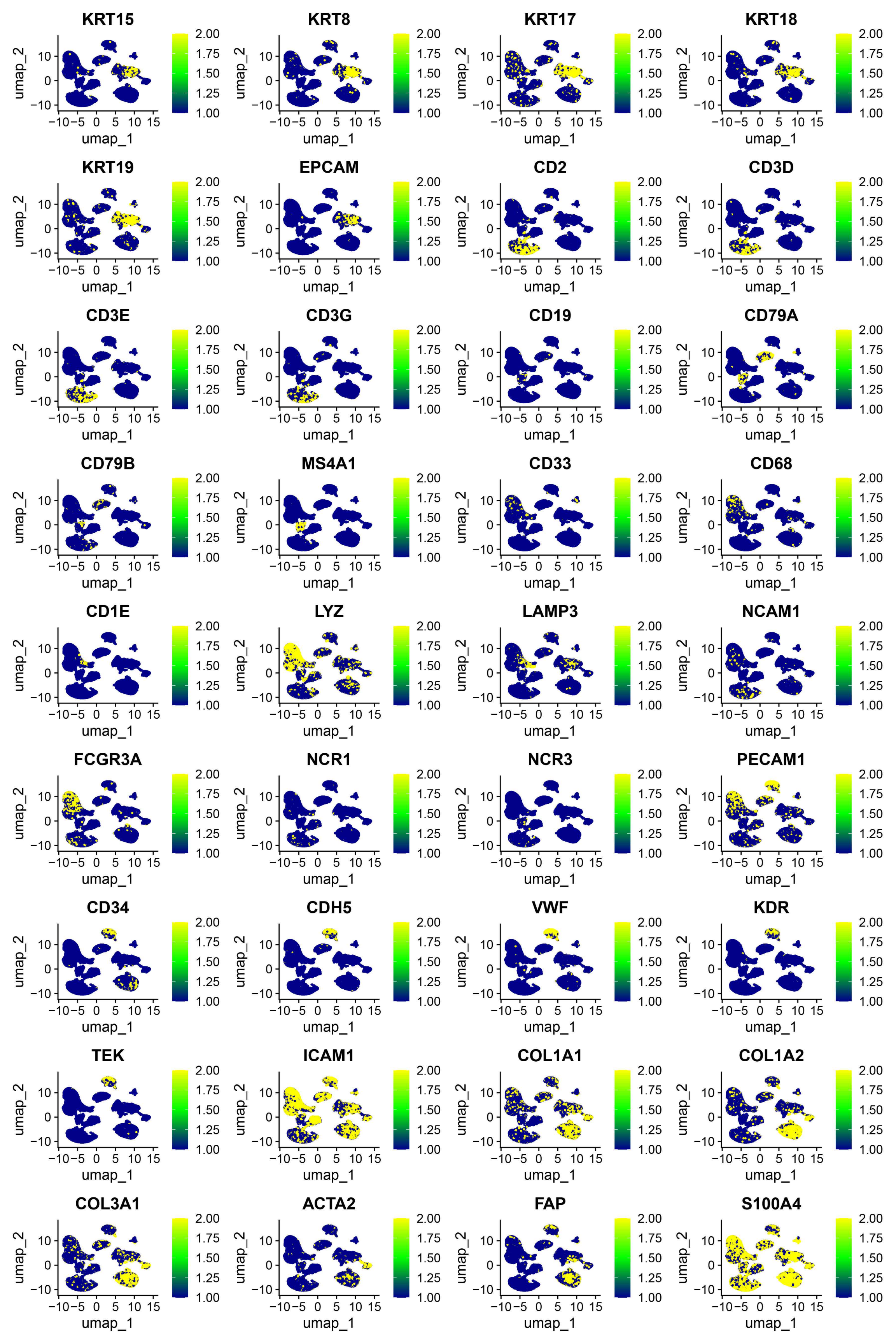

Supplement: Supplementary file 1 — Supplementary material 1 [file 12967_2026_8470_MOESM1_ESM.pdf]

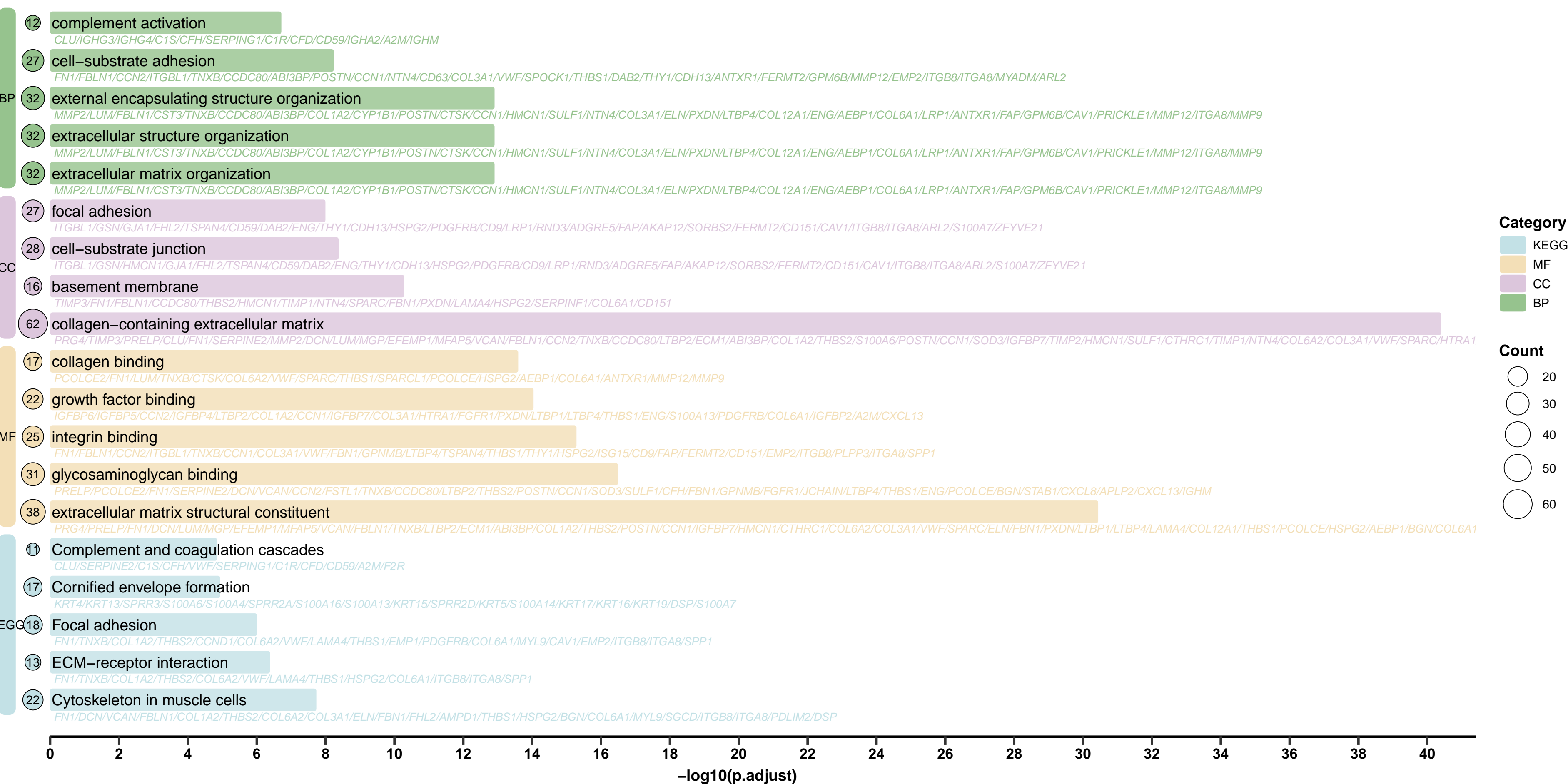

Supplement: Supplementary file 2 — Supplementary material 2 [file 12967_2026_8470_MOESM2_ESM.pdf]

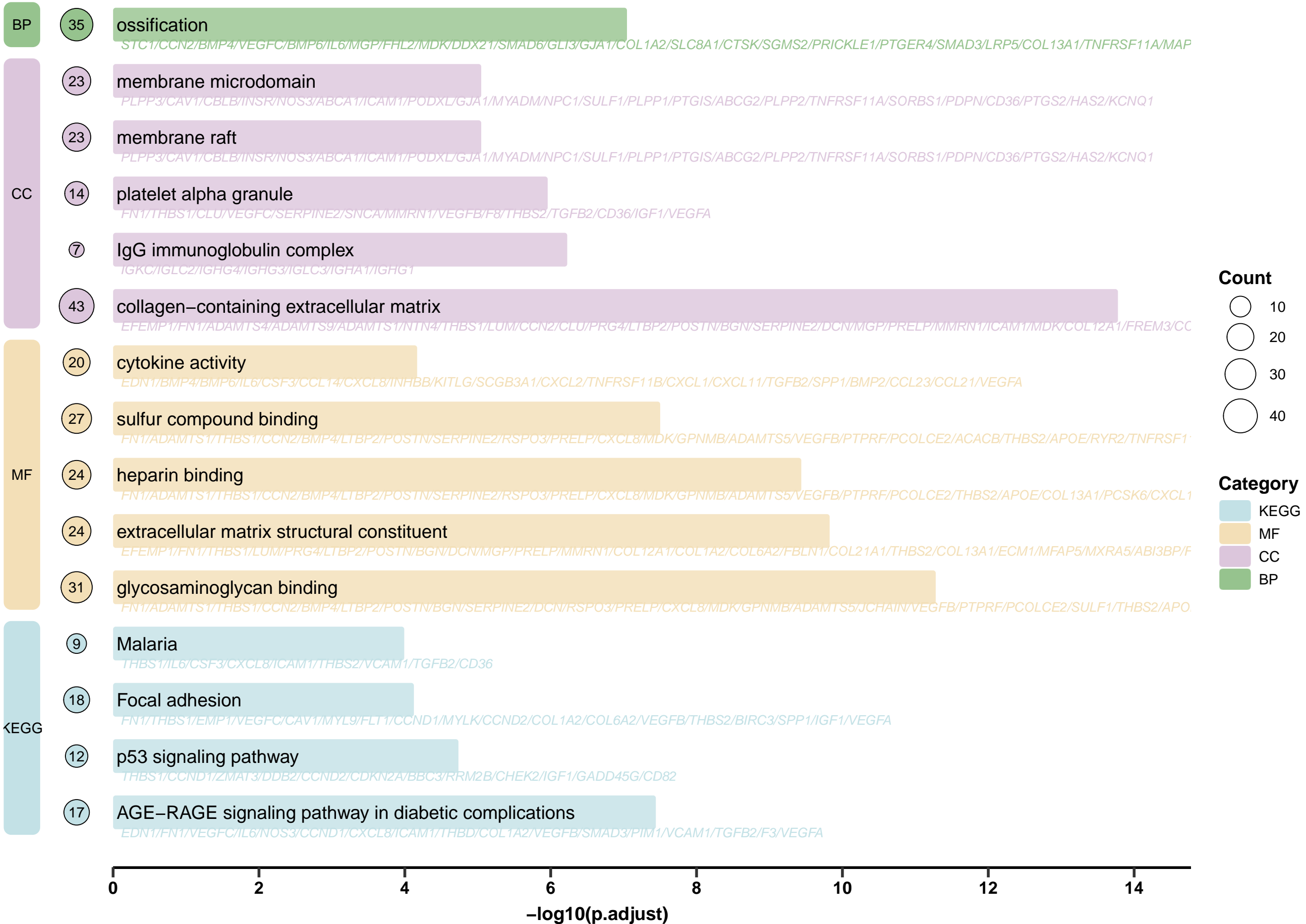

Supplement: Supplementary file 3 — Supplementary material 3 [file 12967_2026_8470_MOESM3_ESM.pdf]

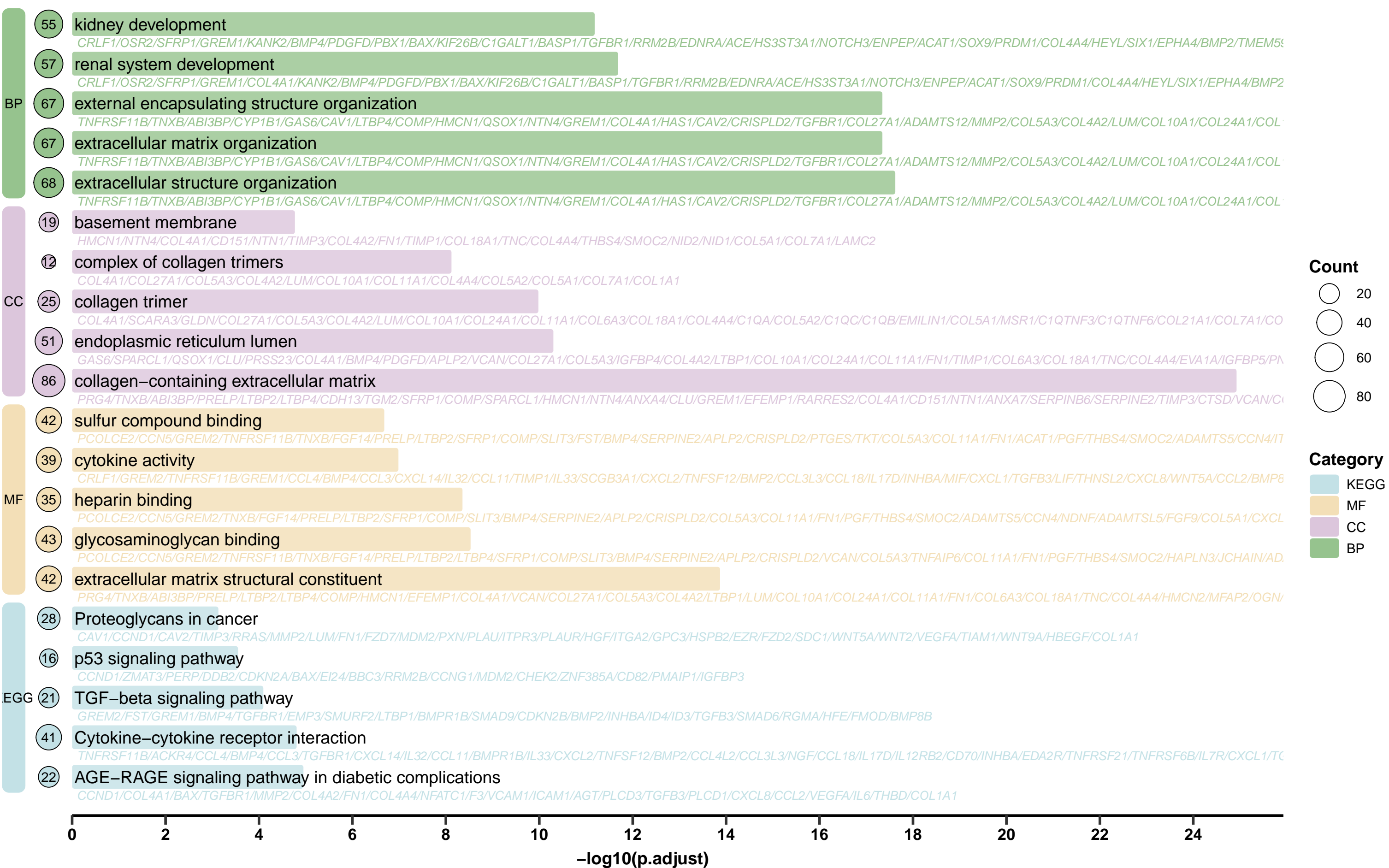

Supplement: Supplementary file 4 — Supplementary material 4 [file 12967_2026_8470_MOESM4_ESM.pdf]

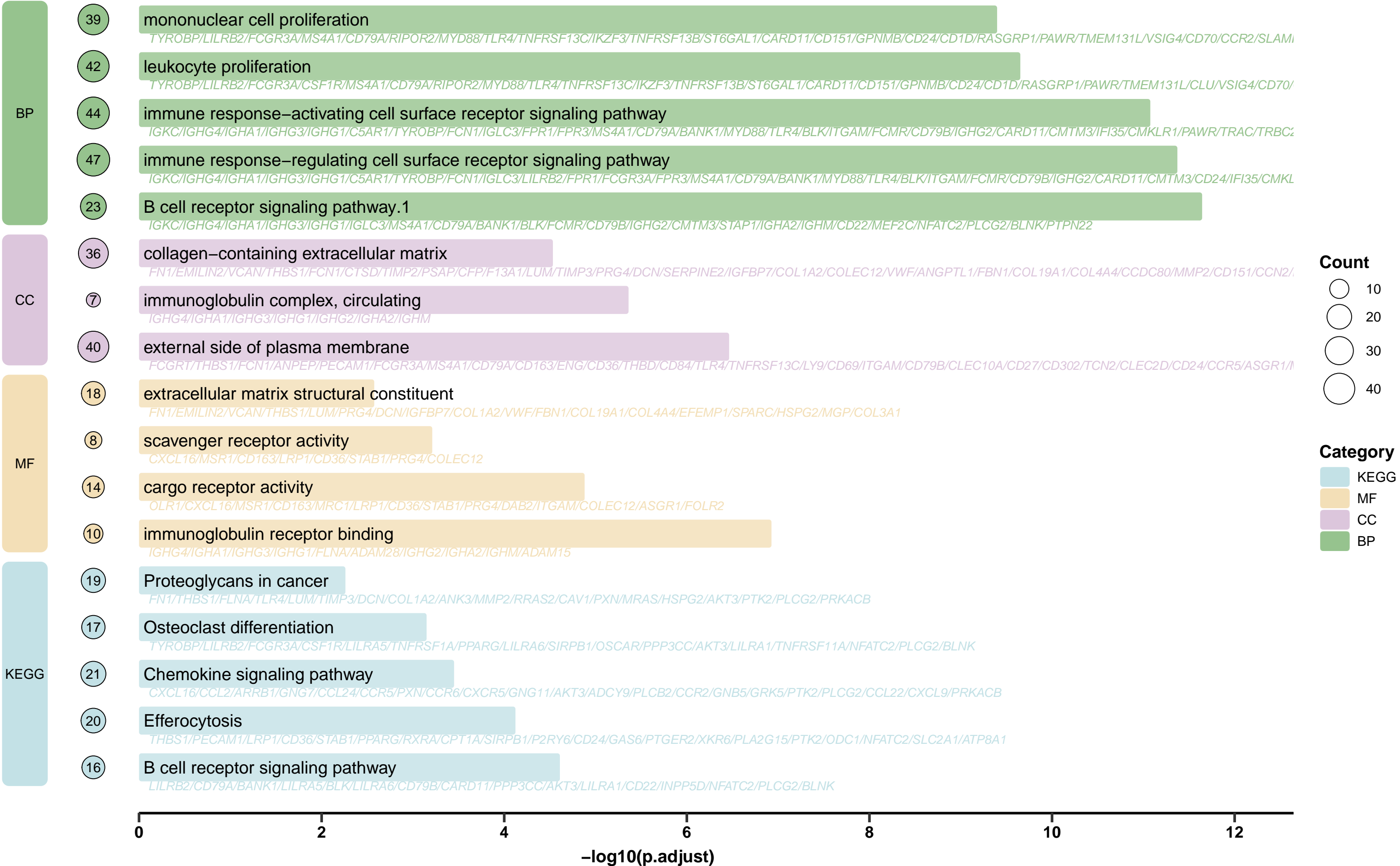

Supplement: Supplementary file 5 — Supplementary material 5 [file 12967_2026_8470_MOESM5_ESM.pdf]

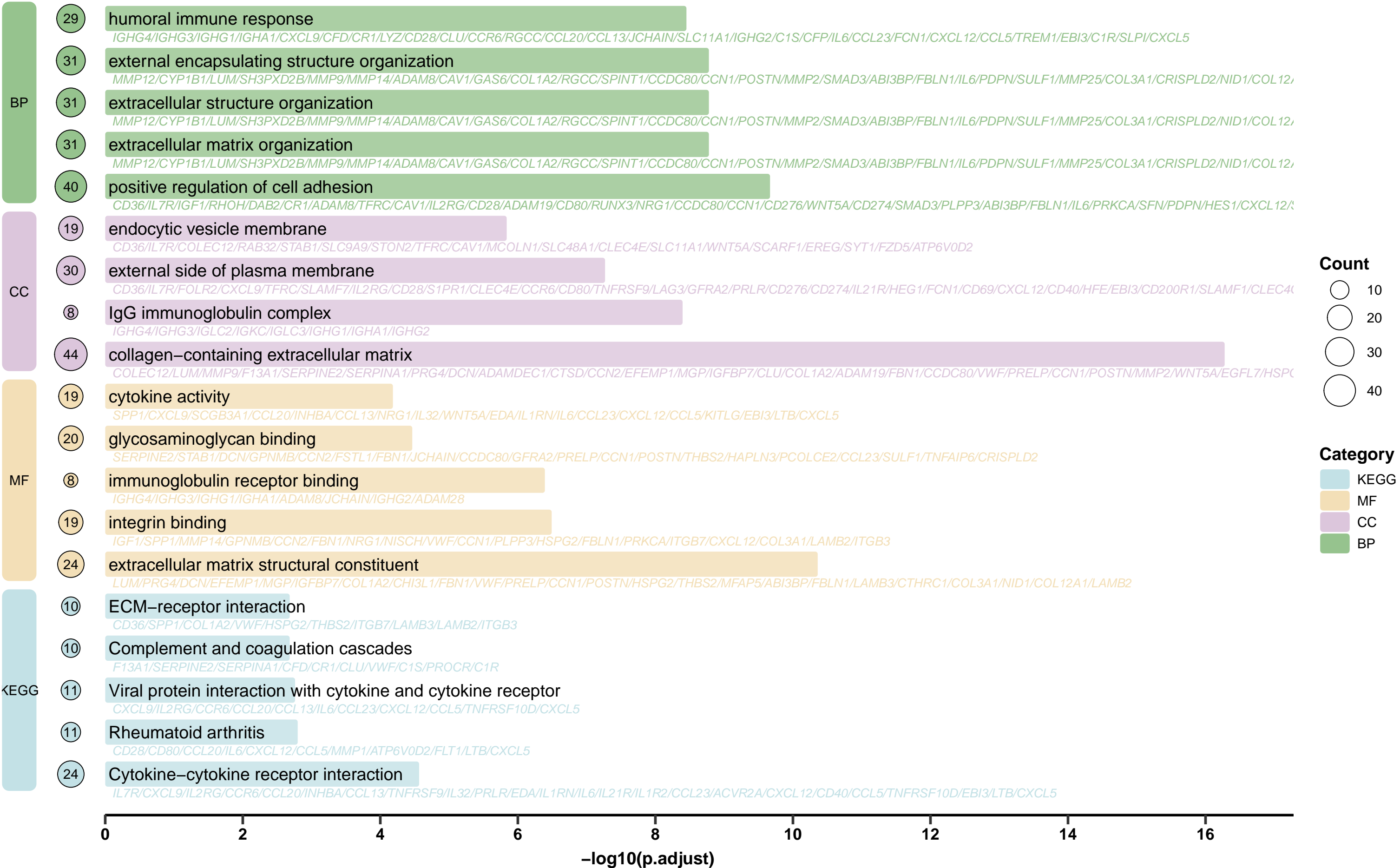

Supplement: Supplementary file 6 — Supplementary material 6 [file 12967_2026_8470_MOESM6_ESM.pdf]

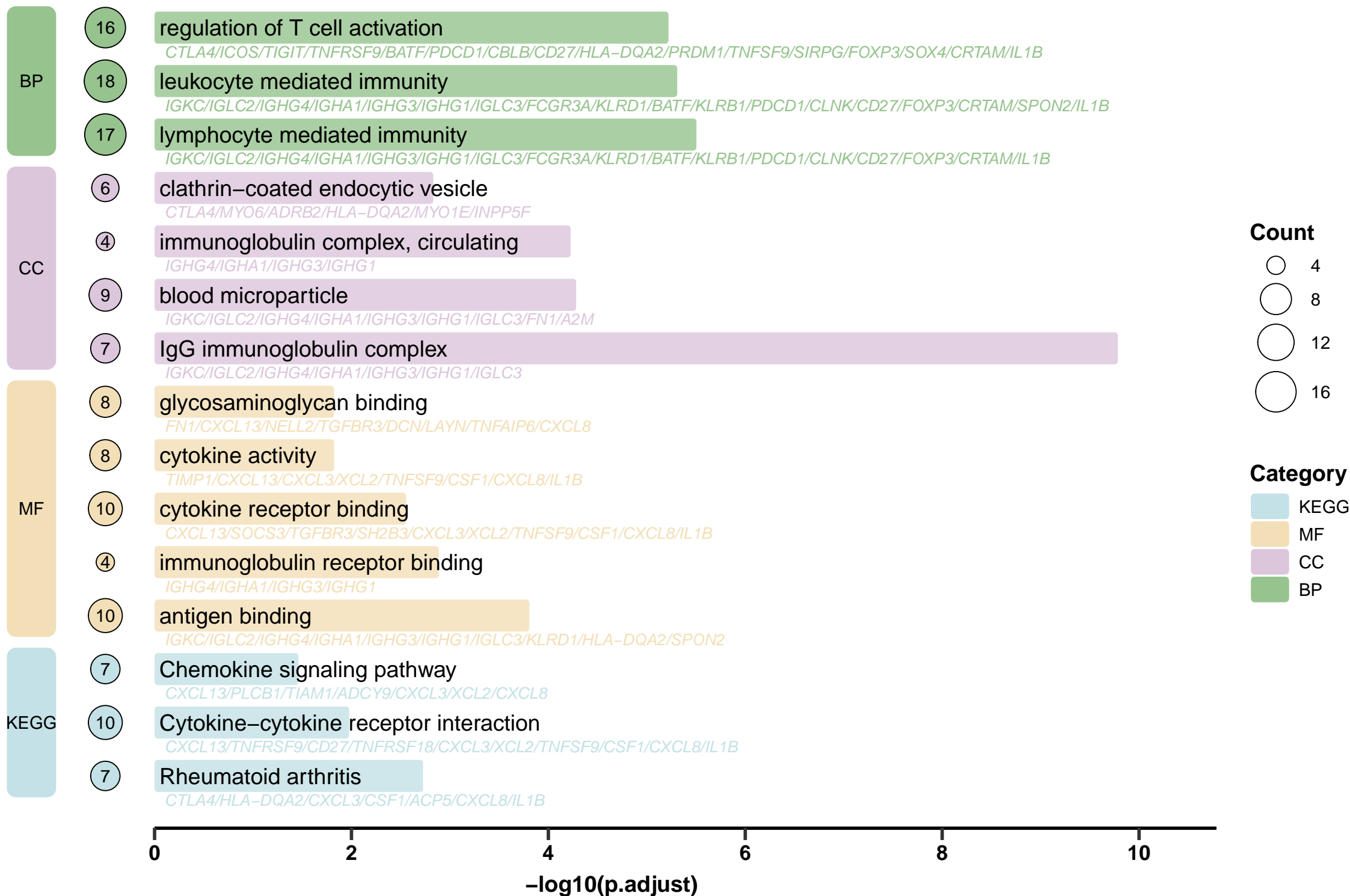

Supplement: Supplementary file 7 — Supplementary material 7 [file 12967_2026_8470_MOESM7_ESM.pdf]

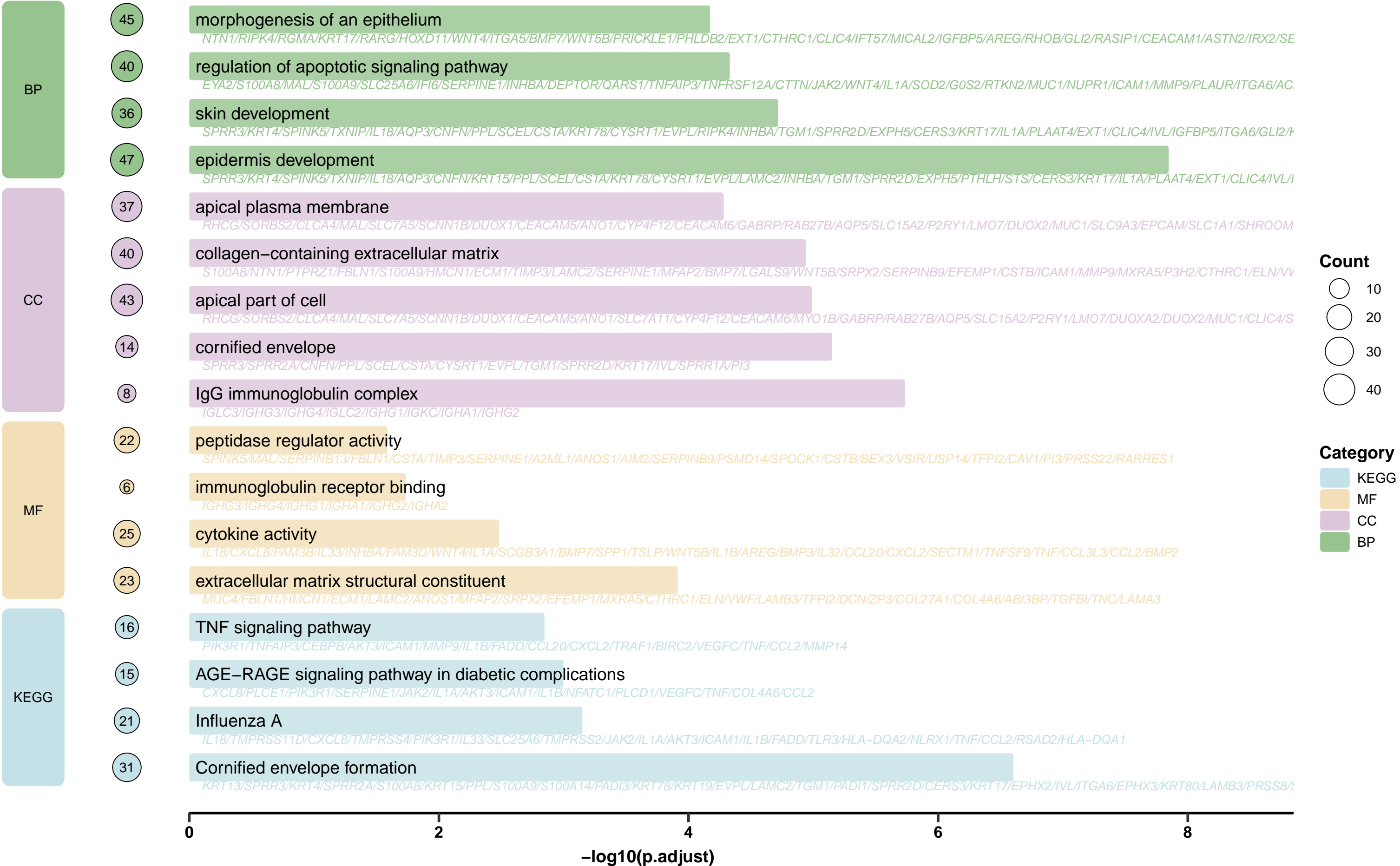

Supplement: Supplementary file 8 — Supplementary material 8 [file 12967_2026_8470_MOESM8_ESM.pdf]
