# Supplementary material for: ACOT9, a mitochondrial metabolism-related gene, promotes ROS-associated epithelial remodeling in laryngeal squamous cell carcinoma
Source: J Transl Med. 2026 Jun 24;24:917. doi: 10.1186/s12967-026-08470-x (PMC13374139; doi:10.1186/s12967-026-08470-x)
Supplement: Supplementary file 9 — Supplementary material 9 [file 12967_2026_8470_MOESM9_ESM.docx]

**Supplementary Figure Legends**

**Supplementary Fig. S1. Identification of single-cell subpopulations based on established marker genes.**

**Supplementary Fig. S2. GO and KEGG enrichment analyses of B cells.**

**Supplementary Fig. S3. GO and KEGG enrichment analyses of endothelial cells.**

**Supplementary Fig. S4. GO and KEGG enrichment analyses of fibroblasts.**

**Supplementary Fig. S5. GO and KEGG enrichment analyses of myeloid cells.**

**Supplementary Fig. S6. GO and KEGG enrichment analyses of NK cells.**

**Supplementary Fig. S7. GO and KEGG enrichment analyses of T cells.**

**Supplementary Fig. S8. GO and KEGG enrichment analyses of epithelial cells.**
